# Weakly ionized gold nanoparticles amplify immunoassays for ultrasensitive point-of-care sensors

**DOI:** 10.1126/sciadv.adn5698

**Published:** 2024-07-10

**Authors:** Jiangjiang Zhang, Fengli Chai, Jia’an Li, Saijie Wang, Shuailong Zhang, Fenggang Li, Axin Liang, Aiqin Luo, Dou Wang, Xingyu Jiang

**Affiliations:** ^1^ Guangdong Provincial Key Laboratory of Advanced Biomaterials, Shenzhen Key Laboratory of Smart Healthcare Engineering, Department of Biomedical Engineering, Southern University of Science and Technology, No. 1088 Xueyuan Road, Nanshan District, Shenzhen, Guangdong 518055, P. R. China.; ^2^ Key Laboratory of Molecular Medicine and Biotherapy, the Ministry of Industry and Information Technology, School of Life Science, Beijing Institute of Technology, Beijing 100081, P. R. China.; ^3^ School of Mechatronical Engineering, Beijing Institute of Technology, Beijing 100081, P. R. China.; ^4^ Australian Institute for Bioengineering and Nanotechnology, The University of Queensland, Brisbane, QLD 4072, Australia.

## Abstract

Gold nanoparticle–based lateral flow immunoassays (AuNP LFIAs) are widely used point-of-care (POC) sensors for in vitro diagnostics. However, the sensitivity limitation of conventional AuNP LFIAs impedes the detection of trace biomarkers. Several studies have explored the size and shape factors of AuNPs and derivative nanohybrids, showing limited improvements or enhanced sensitivity at the cost of convenience and affordability. Here, we investigated surface chemistry on the sensitivity of AuNP LFIAs. By modifying surface ligands, a surface chemistry strategy involving weakly ionized AuNPs enables ultrasensitive naked-eye LFIAs (~100-fold enhanced sensitivity). We demonstrated how this surface chemistry–amplified immunoassay approach modulates nanointerfacial bindings to promote antibody adsorption and higher activity of adsorbed antibodies. This surface chemistry design eliminates complex nanosynthesis, auxiliary devices, or additional reagents while efficiently improving sensitivity with advantages: simplified fabrication process, excellent reproducibility and reliability, and ultrasensitivity toward various biomarkers. The surface chemistry using weakly ionized AuNPs represents a versatile approach for sensitizing POC sensors.

## Teaser

Surface chemistry–mediated nanointerfacial bindings enhance nanomaterial immunoassays rather than the physical size and shape.

## INTRODUCTION

Point-of-care (POC) sensors, particularly gold nanoparticle–based lateral flow immunoassays (AuNP LFIAs), have emerged as robust and practical tools in disease diagnosis, home health care, and pathogen/virus/drug/pesticide residue testing (*1*–*9*). For instance, the COVID-19 pandemic had a devastating impact worldwide. Nucleic acid testing using quantitative real-time polymerase chain reaction (qPCR) is considered the gold standard for identifying infected individuals. However, it necessitates sterile conditions, skilled technicians, and auxiliary equipment. The entire diagnostic process from sample collection to qPCR analysis can take several days when conducted in rural areas or underdeveloped countries. As a valuable complement, AuNP LFIA–based home self-tests offer an efficient primary diagnosis with a naked-eye readout signal that eliminates the risks associated with large gatherings and contributes to epidemic prevention efforts (*10*–*12*).

LFIAs are portable, user-friendly, cost-effective, and time-saving (testing time, ≤15 min). Regrettably, conventional AuNP LFIAs exhibit lower sensitivity compared to enzyme-linked immunosorbent assays (ELISAs) and chemiluminescent/electrochemiluminescence (CL/ECL) immunoassays (*13*–*15*). This limited sensitivity hampers their progression in detecting high-value biomarkers present at trace levels (at pg/ml), such as procalcitonin (PCT)/interleukin inflammatory factors for sepsis, cardiac troponin for myocardial diseases, or microtubule-associated protein tau for neurodegenerative disorders. To enhance sensitivity of AuNP LFIAs, various strategies including AuNP-based nanohybrids (*16*–*21*), inorganic/organic quantum dots/fluorescent beads (*22*–*26*), upconversion nanoparticles (*27*, *28*), photothermal contrast (*29*, *30*), enzyme/nanozyme-linked amplification (*9*, *31*–*33*), Cas endonuclease–mediated amplification (*34*, *35*), loop-mediated isothermal amplification (*36*, *37*), surface-enhanced Raman scattering (SERS) technique (*38*, *39*), and spin-enhanced nanodiamond technique (*40*) have been developed to complement traditional LFIAs. We have successfully developed ultrasensitive LFIAs on the basis of fluorescent metal–AIEgen frameworks (MAFs) (*7*, *41*). Because of high quantum yield, mesoporous morphology, and strong physicochemical affinity to antibodies (Abs), the limit of detection (LOD) for MAF LFIA is <1 pg/ml. In addition to improving sensitivity, these enhancements require supplementary operations, reagents, or devices, or they are based on intricate fabrication. However, achieving increased sensitivity without sacrificing convenience and cost/time efficiency remains challenging yet highly desirable.

In this study, we have developed a surface chemistry strategy for weakly ionized AuNP LFIAs, resulting in heavily enhanced sensitivity while maintaining convenience and cost/time efficiency. The key component of AuNP LFIA is the Ab-labeled AuNP complex (Ab@AuNP). Typically, commercially available and laboratory-used AuNPs in LFIA are coated with citrate ligands (Cit-AuNPs). The ligands regulate the surface chemistry of AuNPs to determine their specific physicochemical properties (*42*–*51*). Cit becomes Cit^3−^ under neutral pH, forming a potent electrostatic nanointerface of Cit-AuNPs, i.e., the strongly ionized AuNPs. Most Abs have an isoelectric point (pI) around pH 8. To prevent charge interaction–induced aggregation of Cit-AuNPs and Abs, the buffer pH is adjusted to ~8.5 when preparing Ab-labeled Cit-AuNP (Ab@Cit-AuNP). Ab binds to the surface of AuNP through physicochemical adsorption. However, the intense electrostatic layer mediated by Cit ligands may repel Abs and weaken their binding affinity onto the surface of AuNP. This phenomenon could be one of the key factors determining the sensitivity observed in traditional Cit-AuNP LFIAs.

**Fig. 1.**
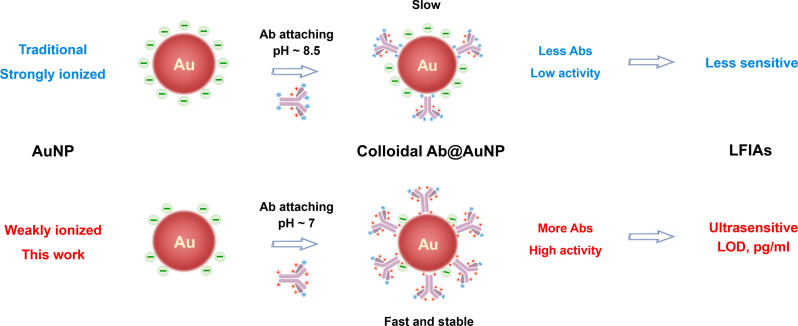
Schematic illustration. Schematic illustration of the proposed weakly ionized AuNPs for straightforward but ultrasensitive LFIAs.

**Fig. 2.**
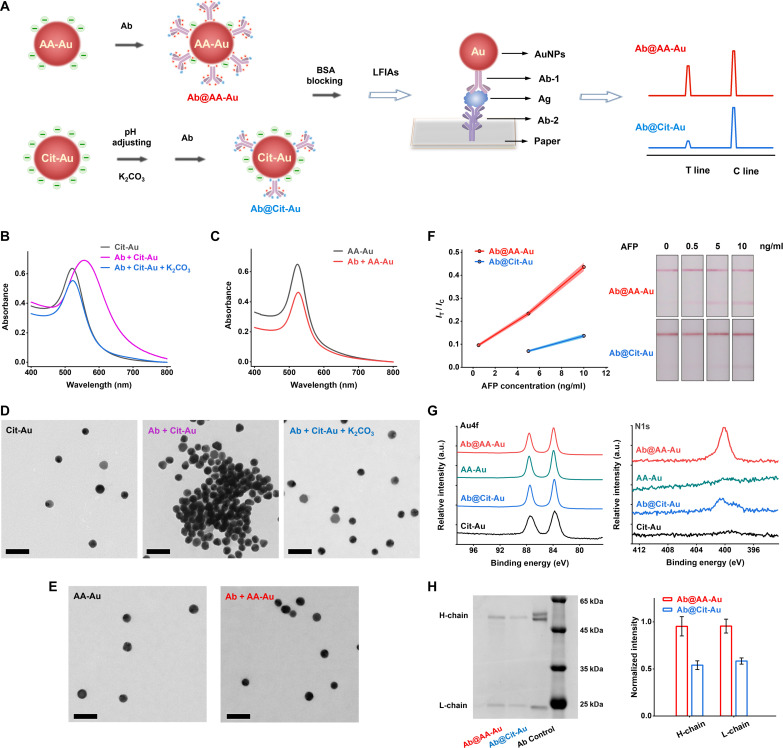
Comparisons between weakly ionized AA-AuNPs and traditional strongly ionized Cit-AuNPs. (**A**) Schematic illustration of strongly ionized Cit-AuNP LFIA and weakly ionized AA-AuNP LFIA. (**B** and **C**) The absorption spectra of Cit-AuNPs and AA-AuNPs before and after incubation with Ab. (**D**) Transmission electron microscopy (TEM) images of Cit-AuNPs, Cit-AuNPs after incubation with Ab, and Cit-AuNPs after incubation with Ab using K_2_CO_3_ to alter the pH. Scale bars, 100 nm. (**E**) TEM images of AA-AuNPs and AA-AuNPs after incubation with Ab. Scale bars, 100 nm. (**F**) The intensity ratio (*I*_T_/*I*_C_) curves of AA-AuNP LFIAs and traditional Cit-AuNP LFIAs via different concentrations of AFP. (The colored error bars represent three different replicates.) Right: The corresponding photographs of AA-AuNP LFIA and traditional Cit-AuNP LFIA strips. (**G**) The Au4f and N1s XPS binding energy profiles of AA-AuNPs and Cit-AuNPs before and after incubation with Ab, respectively. (**H**) The gel image of the detached protein from collected Ab@AA-AuNPs and Ab@Cit-AuNPs complexes after SDS-PAGE analysis (stained with Coomassie brilliant blue). Right: The normalized intensity of the heavy chain (H-chain) and light chain (L-chain) of the detached Abs (the error bars represent more than three different replicates). a.u., arbitrary units.

To increase sensitivity and maintain convenience and cost/time efficiency, we formulated the surface chemistry of AuNPs featuring a loose electrostatic nanointerface, i.e., weakly ionized AuNPs, to promote Ab adsorption (Fig. 1). We synthesized ascorbic acid–coated AuNPs (AA-AuNPs) and investigated the AA-AuNP LFIA. AA is altered to AA^−^ in a neutral pH environment. We classify AA-AuNPs as the proposed weakly ionized AuNPs compared to strongly ionized Cit-AuNPs. AA ligands create a loose electrostatic layer that prevents aggregation caused by charge interaction during incubation with Abs. Regulating buffer pH is unnecessary for the production of Ab@AA-AuNP. This approach simplifies the synthesis process of AuNP LFIAs, thereby offering convenience and time/cost efficiency enhancements (Fig. 2A). In addition, AA-AuNPs benefit from regulated ligands at the nanointerfacial level, which delivers a stronger binding affinity of Ab than Cit-AuNPs. This conceptual design of weakly ionized AuNPs is supported by x-ray photoelectron spectroscopy (XPS) tests, SDS–polyacrylamide gel electrophoresis (SDS-PAGE) analysis, and molecular dynamics (MD) simulations. Under identical conditions and comparable sizes between AA-AuNPs and Cit-AuNPs, LFIA based on AA-AuNPs exhibits greater sensitivity than traditional LFIA using Cit-AuNPs. Moreover, this enhanced sensitivity surpasses that achieved using fluorescent or CL LFIA approaches when detecting various blood biomarkers such as α-fetoprotein (AFP), C-reactive protein (CRP), and PCT. Given its high sensitivity performance, we further explored virus detection capabilities using AA-AuNP LFIAs. AA-AuNP LFIAs successfully distinguished between wild-type severe acute respiratory syndrome coronavirus 2 (SARS-CoV-2) and the B.1.1.7 mutant strain by comparing the signal intensity of the T lines from two distinct antigen (Ag) sites. Leveraging weakly ionized AuNPs as a surface chemistry strategy for sensitizing immunoassays holds great promise in developing ultrasensitive POC sensors.

## RESULTS

We synthesized AA-AuNPs by a one-pot reaction. AA solution (1% and 1.5 ml) was rapidly pipetted into the HAuCl_4_ solution (1 mM and 50 ml) under vigorous stirring at 90°C. The solution turned deep red after 20 min. AA is the reducing agent and also the stabilizing ligand. AA-AuNPs are nearly spherical, with a size distribution of 30.4 ± 7.8 nm (fig. S1). The traditional Cit-AuNPs (30.6 ± 6.0 nm) were commercially available for comparison tests. Ligands ionization produces different surface zeta potentials of AuNPs. Under neutral pH, the zeta potential of Cit-AuNPs is −35.4 ± 5.1 mV, while that of AA-AuNPs is −20.5 ± 6.5 mV (fig. S2). AA is less ionized than the strongly ionized Cit ligand (AA^−^ versus Cit^3−^). We defined AA-AuNPs as the weakly ionized AuNPs compared to the strongly ionized Cit-AuNPs. To prepare the Ab@AuNP, we first normalized the concentrations of AA-AuNPs and Cit-AuNPs by diluting them to balance their absorbance at 522 nm (fig. S3). The direct addition of Abs caused the aggregation of strongly ionized Cit-AuNPs (red shift of absorption peak and broadening absorption band, Fig. 2B; purple color, fig. S4; and clustered particles, Fig. 2D), owing to the strong electrostatic interaction between negatively charged Cit-AuNPs and positively charged Abs (Cit: p*K_a_*, 6.40; Ab: pI of pH ~ 8). To prepare the colloidal Ab@Cit-AuNP, we adjusted the pH by adding bases such as K_2_CO_3_. When the pH was above 8, the total charge of Ab turned slightly negative while carrying positive charges locally. The electrostatic interaction–caused aggregation of negatively charged Cit-AuNPs was abolished. The product colloidal Ab@Cit-AuNP shows a decreasing absorbance without apparent red shift or clustering behavior (Fig. 2, B and D). The adsorption of Abs reduced the zeta potential of Cit-AuNPs (fig. S5). For the weakly ionized AA-AuNPs, the direct addition of Abs at neutral pH was straightforward and efficient in obtaining the colloidal Ab@AA-AuNP. The absorbance decrease was observed without apparent red shift, color change, or clustering behavior (Fig. 2, C and E, and fig. S4), similar to the colloidal Ab@Cit-AuNP. The weak ionization property of AA ligands avoided the charge interaction–mediated aggregation of AA-AuNPs when incubated with Abs. At the same time, the attachment of Abs at neutral pH reversed the zeta potential of AA-AuNPs. The colloidal Ab@AA-AuNP showed a positive charge (fig. S5).

Using the collections of two colloidal Ab@AuNPs, we compared their performance on LFIAs. Under the conditions of test (T) line (capture Ab, 1 mg/ml) and control (C) line (anti-Ab, 0.5 mg/ml), we optimized the label Ab concentration of 10 μg/ml to achieve a high color signal of T line (fig. S6). We fabricated the weakly ionized AA-AuNP–based strips and the strongly ionized Cit-AuNP–based strips to test the responses to different concentrations of AFP. We measured the intensity ratio of T/C (*I*_T_/*I*_C_) to assess their sensitivities. At high concentrations (5 and 10 ng/ml), AA-AuNP LFIA has over three times higher *I*_T_/*I*_C_ signal than Cit-AuNP LFIA (Fig. 2F). At a low concentration of 0.5 ng/ml, Cit-AuNP LFIA was not recognized as an obvious color signal of the T line. However, AA-AuNP LFIA showed a clear color signal readable by the naked eye (Fig. 2F). AA-AuNP LFIA is more sensitive than the traditional Cit-AuNP LFIA. The weakly ionized AA-AuNPs sensitize Ab/Ag recognition–mediated immunoassay. To understand this sensitization, we did XPS and SDS-PAGE tests to analyze the two different Ab@AuNP complexes (*7*, *52*). Au4f profiles have no changes for AA-AuNPs/Cit-AuNPs before and after incubation with Abs (Fig. 2G and fig. S7). The present binding energy peaks in N1s profiles indicate the proteins adsorbed onto the surfaces of AuNPs because the N element is assigned to proteins while AA and Cit do not contain N atoms. SDS-PAGE analysis further verified that the protein is Ab (typical bands of heavy chain ~50 kDa and light chain ~25 kDa; Fig. 2H and fig. S8). According to the relative quantification, the amount of Ab adsorbed on surfaces of AA-AuNPs is higher (about two times) than that of Cit-AuNPs. The weakly ionized AA-AuNPs are advantageous for the physicochemical adsorption of Ab compared to the traditional strongly ionized Cit-AuNPs.

**Fig. 3.**
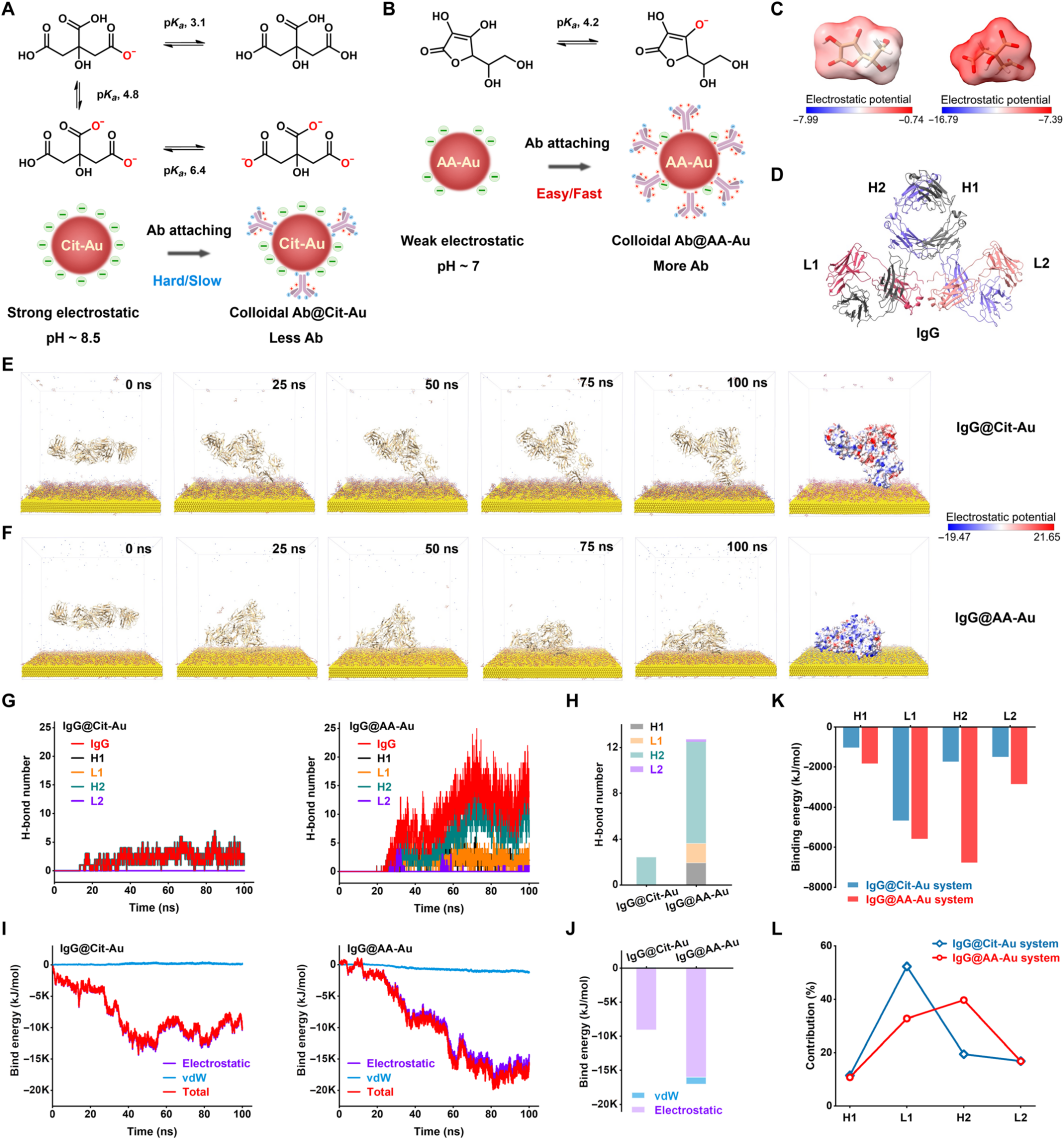
Theoretical analysis based on MD simulations and calculations. (**A** and **B**) The ionization of Cit/AA and the schematic illustration of Ab attaching on Cit-AuNP and AA-AuNP. (**C**) The electrostatic potential distribution of Cit^3−^ and AA^−^. (**D**) The cartoon image of IgG molecule. (**E** and **F**) The snapshots of IgG molecules on the Cit-Au and AA-Au surfaces at different simulation times. The rightmost images are the corresponding electrostatic potential of IgG molecules on Au surfaces. For a clear view, water molecules and nonpolar H atoms are hidden. (**G**) The time-related curves of H-bond number between the IgG molecule and the Cit-Au/AA-Au surface. (**H**) The average H-bond number of IgG subunits (H1, L1, H2, and L2) at the last 10-ns simulation. (**I**) The time-related energy curves of the IgG@Cit-Au system and IgG@AA-Au system during the simulation. (**J**) The average van der Waals (vdW) force and electrostatic interaction between IgG molecule and Cit-Au/AA-Au surface at the last 10-ns simulation. (**K**) The average energy between IgG subunits (H1, L1, H2, and L2) and Cit-Au/AA-Au surface at the last 10-ns simulation. (**L**) The related contribution curves of different IgG subunits.

To further explore the molecular mechanism of the sensitization of AA-AuNPs, we conducted MD simulations to study the nanointerfacial interactions between the Ab [immunoglobulin G (IgG)] and ligand-coated Au surface. A planar Au(111) surface (~20 nm by 20 nm) was built to simplify the simulation instead of the spherical nanoparticle (fig. S9A). Cit and AA ionization are presented in Fig. 3 (A and B). Under the optimal conditions, Cit/AA turns to Cit^3−^/AA^−^. They were loaded on the Au surface with a density of 2/nm^2^ at pH 8.5 and 7 on the basis of the conditions for preparing the colloidal Ab@AuNP, respectively. Figure 3C shows the electrostatic potential distribution of Cit^3−^ and AA^−^. The ring structure of AA favors forming a stacking interaction, like the π-π stacking. The binding energy of the AA-Au system is over two times higher than that of the Cit-Au system (fig. S9, B and C). The AA-Au system is more stable than the Cit-Au system. To simulate the binding process, we placed the IgG molecule above the prebalanced Au surface (centroid distance, ~7.3 nm; fig. S10). When the simulation started, IgG gradually attached to the Au surface (Fig. 3, E and F). The centroid distance between the IgG and AA-Au surface decreased rapidly and was equilibrated at nearly 60 ns (change, ~3.8 nm; fig. S11A). However, the centroid distance for the IgG@Cit-Au system slightly increased (~0.3 nm). We calculated the contact area to show the interaction strength (fig. S11B). When the centroid distance decreased to 5.2 nm (~20 ns), the contact happened in the IgG@AA-Au system. The maximum value is 29.8 nm^2^. For the IgG@Cit-Au system, the contact area (4.6 nm^2^) was ~6.5 times smaller than the IgG@AA-Au system. The snapshots and video of MD simulations show that IgG stands leaning on the Cit-Au surface, while IgG lies on the AA-Au surface (Fig. 3, E and F; movies S1 and S2). The decreased centroid distance and a larger contact area indicate a stronger binding interaction between IgG and the AA-Au surface in contrast to the Cit-Au surface.

IgG contains two heavy chains and two light chains (H1, L1, H2, and L2; Fig. 3D). We analyzed the detailed interactions between these subunits and the Au surface. To evaluate the simulation accuracy, we recorded the root mean square deviation (RMSD) of the backbone atoms versus their initial ground state, the root mean square fluctuation (RMSF) of the residues’ movement, and the subunits’ gyration radius (Rg). The RMSD equilibrium point of IgG and different subunits is about 60 ns/20 ns for the IgG@Cit-Au system/IgG@AA-Au system (fig. S12). IgG on the AA-Au surface achieves a balanced conformation faster than on the Cit-Au surface. RMSF is used to evaluate the freedom of the residues’ movement and reflects structural flexibility. Subunits H1/H2/L2 of the IgG@Cit-Au system and H1/H2 of the IgG@AA-Au system show relatively higher RMSF values (fig. S13), which suggests that these subunits may be affected by Au surfaces. Rg reflects the compactness of proteins. For the IgG@Cit-Au system, the Rg of subunits H1 and L2 reduced markedly (fig. S14). For the IgG@AA-Au system, the remarkable reduction is for the H1 and H2. The Rg change is consistent with the centroid distance and contact area changes (the smaller distance, the larger contact area, and the smaller Rg). Rg reduction reveals the potential tight interaction between these subunits and the Au surface. When IgG interacts with the Cit-Au surface, subunits H1 and L2 dominate the binding interaction. As for the AA-Au surface, subunits H1 and H2 take the majority instead. This discrepancy indicates that ligand-mediated surface chemistry plays an essential role in the binding process and has a propensity to the different subunits when IgG interacts with the Au surface. The strongly ionized Cit-Au surface processing a dense electrostatic layer shows the affinity to both H and L subunits. However, the weakly ionized AA-Au surface processing a loose electrostatic layer shows the preferred affinity to the H subunit.

We monitored the H-bond number and energy profiles of the IgG@Au systems to analyze the driving force of binding interaction. Cit ligand has three negative charges. Only a few H-bonds (~2.4 per) were formed in the IgG@Cit-Au system (Fig. 3G). AA ligand has one negative charge and contains multiple hydroxyl groups. As the contact area increased in the IgG@AA-Au system, the H-bond formed and increased rapidly. The average H-bond number was 12.8 at the last 10-ns simulation (over five times higher than the IgG@Cit-Au system). Figuring the detailed contribution of these H-bond, subunit H2, contributed mostly about 70% (Fig. 3H). While in the IgG@Cit-Au system, the contribution of subunit H2 was 100%. The H-bond analysis correlates with the RMSF and Rg results. The energy profiles show that the electrostatic interaction far outweighs the van der Waals force when IgG attaches to the Cit-Au/AA-Au surface (Fig. 3I). The binding energy between IgG and AA-Au surface is about two times higher than that of the Cit-Au surface (Fig. 3J), indicating that IgG@AA-Au is more stable than IgG@Cit-Au. The energy profiles of different subunits further reveal the detailed binding sites (Fig. 3K and figs. S15 and S16). Subunits L1 and H2 offer the predominance function for the IgG attachment. Counting the contributions, L1 was the major contributor to the binding interaction in the IgG@Cit-Au system (>50%). For the IgG@AA-Au system, the major contributor was replaced with H2. Owing to the big difference in molecular weight, the light chain subunit is more responsive to external influence than the heavy chain subunit. When the light chain subunit involves the binding interaction with the Au surface and plays a key role in this process, their biochemical functions, such as Ag recognition, would certainly be weakened. The above MD simulation results suggest that IgG in the IgG@AA-Au system is more stable and has relatively higher activity for the Ab-Ag immune recognition than in the IgG@Cit-Au system. Besides the MD simulation, the XPS tests and SDS-PAGE analysis have proved that more Abs are adsorbed on surface of AA-AuNPs than Cit-AuNPs (Fig. 2, G and H). To compare the bioactivity of Abs on the surface of AuNPs, we prepared the AA-AuNPs and Cit-AuNPs modified with two different Abs (Ab1 and Ab2) for the immuosandwich aggregation tests (fig. S17, A and B). When target Ag binds with Ab1 and Ab2, the higher activity of Abs produces a stronger binding interaction, leading to more aggregation between Ab1@AuNPs and Ab2@AuNPs. The mixture of Ab1@AA-AuNPs and Ab2@AA-AuNPs showed more serious aggregation than Ab1@Cit-AuNPs and Ab2@Cit-AuNPs (fig. S17, C to G). The immuosandwich aggregation tests reveal a higher activity of Abs on the surface of the weakly ionized AA-AuNPs compared to the strongly ionized Cit-AuNPs. These experimental tests are consistent with the MD simulations. We tested the nanobody to evaluate whether it applies to the enhanced AA-AuNP LFIA. As the minimum Ab, the nanobody only consists of the variable domain of classic Ab’s H chain. The synthesis process was the same as the above, which used classic Abs. Losing the H chain’s constant domain, the nanobody cannot bind the anti-Ab on the C line. Because the nanobody is small (molecule mass, ~15 kDa), it will likely be shielded or replaced with the blocking agent BSA. The tight interaction with the Au surface weakens the bioactivity of nanobodies. Nanobody-labeled AA-AuNPs may have low efficiency in binding the target Ag. Checking the tested strips of nanobody-labeled AA-AuNP LFIA, the T and C lines are colorless without any recognizable signal (fig. S18). Thus, compared to the classic Ab, the nanobody is inapplicable to the AA-AuNP LFIAs. On the basis of the MD simulations and the experimental tests, we explained the enhanced sensitivity of the AA-AuNP LFIAs: (i) The weakly ionized AA-AuNPs are more effective than the strongly ionized Cit-AuNP for Ab adsorption. Under the optimal conditions, more Abs adsorbed on the surface of AA-AuNP than Cit-AuNP. (ii) Different subunits of Ab lead to the binding between the Ab and AA-Au/Cit-AuNP surface. Ab in Ab@AA-AuNP has a higher bioactivity than in Ab@Cit-AuNP for Ab-Ag immune recognition.

**Fig. 4.**
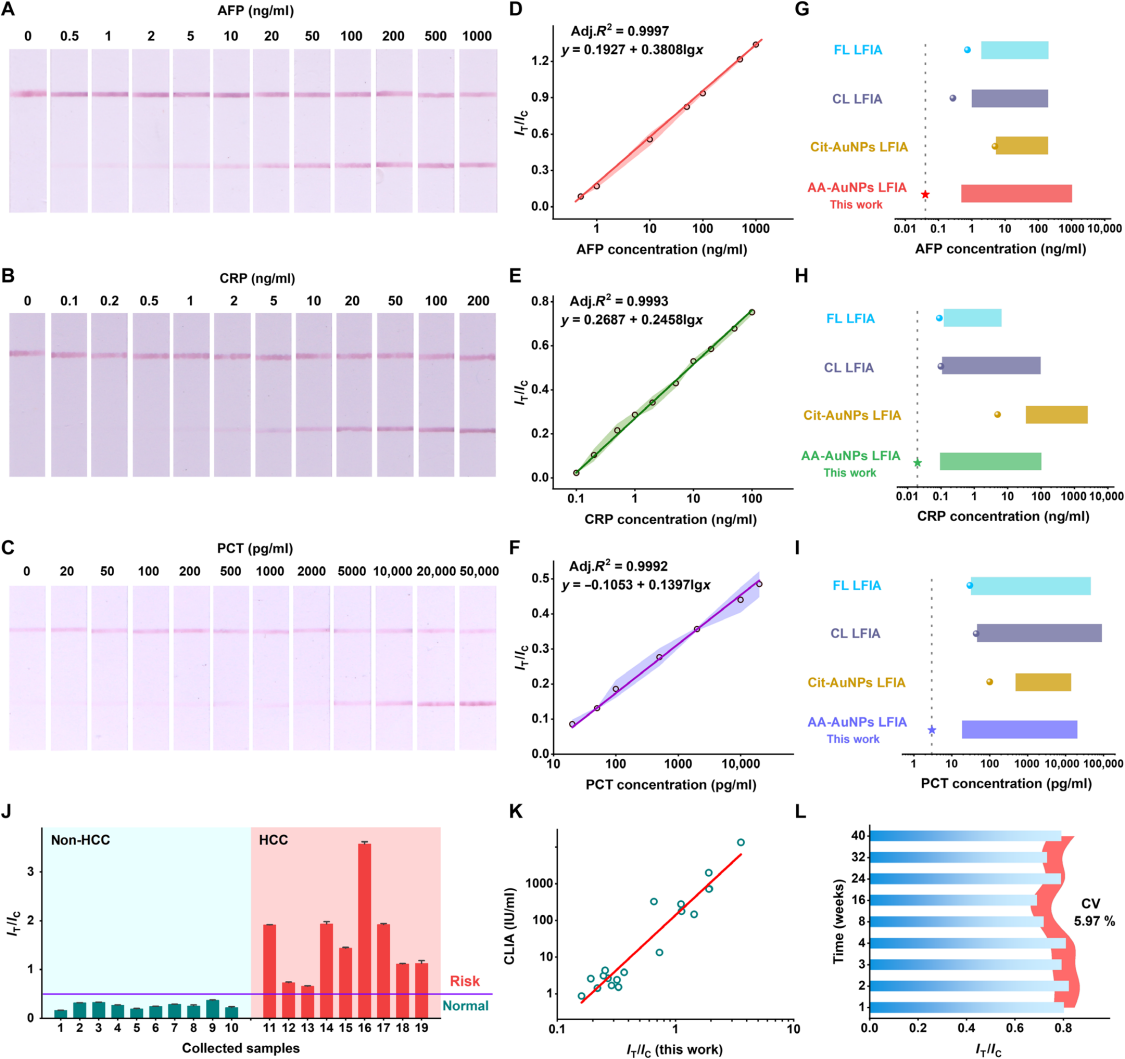
Analytical performance of weakly ionized AA-AuNP LFIAs. (**A** to **C**) The testing strips of AA-AuNP LFIAs responding to different concentrations of AFP, CRP, and PCT, respectively. (**D** to **F**) The correlated linear fitting curves of AFP (0.5 to 1000 ng/ml), CRP (0.1 to 100 ng/ml), and PCT (20 to 20,000 pg/ml), respectively. The colored error bars represent three different replicates. (**G** to **I**) Comparisons of the LOD value (plots) and the linear detection range (bands) of different methods (CL, chemiluminescent; FL, fluorescent; details seen in table S1 of the supporting materials). (**J**) The testing results of 19 clinical samples using the AA-AuNP LFIA (the non-HCC group: 10 samples; the HCC group: 9 samples). The cutoff line represents the threshold value of AFP at 20 ng/ml. (**K**) The correlation analysis between the clinic detection method (CLIA) and the AA-AuNP LFIA (this work). (**L**) The time-related stability testing results of the AA-AuNP LFIA responding to AFP at 100 ng/ml at the same conditions under room temperature (the relevant images of tested strips are seen in fig. S25). The colored error bars represent three different replicates.

We tested different serodiagnostic markers to prove the sensitivity and universality of the weakly ionized AA-AuNP LFIAs. AFP is a biomarker for liver cancer diagnosis. CRP and PCT are inflammatory factors related to many diseases. PCT is a key biomarker for assessing sepsis. Under the optimized conditions, we fabricated the weakly ionized AA-AuNP LFIAs responding to different biomarkers. The testing strips and the concentration-dependent intensity ratio (*I*_T_/*I*_C_) are shown in Fig. 4 (A to C) and fig. S19. The naked-eye detection limit is 0.5 ng/ml for AFP, 1 ng/ml for CRP, and 50 pg/ml for PCT. These visual detection limits meet the clinic diagnosis standards (cutoff values: AFP, 20 ng/ml; CRP, 800 ng/ml; and PCT, 50 pg/ml). The linear detection ranges are well fitted with adjusted *R*^2^ > 0.999 for all testing biomarkers (Fig. 4, D to F). The calculated LOD (3σ/slope) of AA-AuNP LFIAs is 40 pg/ml for AFP, 20 pg/ml for CRP, and 3 pg/ml for PCT. The weakly ionized AA-AuNP LFIAs are more sensitive than traditional strongly ionized Cit-AuNP LFIAs in the literature and this work (figs. S20 to S22). The maximum enhancement is over 10^2^ times (Fig. 4, G to I, and tables S1 and S2). Comparing the LOD and detection range, the developed weakly ionized AA-AuNP LFIAs are superior to the reported fluorescent LFIAs and CL LFIAs (Fig. 4, G to I, and table S1) (*9*, *22*, *25*, *53*–*57*). When taking the LOD value as an index, the straightforward colorimetric AA-AuNP LFIAs are comparable to the complicated SERS LFIAs and nanozyme LFIAs (table S1) (*39*, *58*–*61*). To test the performance in real samples, we collected the clinical serum samples of hepatocellular carcinoma (HCC) and non-HCC patients (table S3) to detect AFP using AA-AuNP LFIAs. First, we tested the standard curve in the spiked serum (fig. S23). According to the cutoff of 20 ng/ml, the testing results from AA-AuNPs LFIAs are positive for all nine HCC samples (containing two weakly positive cases, no.12/13; Fig. 4J and fig. S24). At the same time, the testing results of 10 non-HCC samples are negative. To verify the reliability of AA-AuNP LFIA, we compared it to the clinic CL immunoassay (CLIA). AA-AuNP LFIA correlates highly with the CLIA (adjusted *R*^2^ > 0.9; Fig. 4K). The time-related tests show a variable coefficient (CV) < 6%, indicating good stability of AA-AuNP LFIA after 6 months of storage (Fig. 4L and fig. S25). We tried to synthesize different sizes of AA-AuNPs to test the size effect on LFIAs. Obtaining uniform large AA-AuNPs (~50 nm) was hard because of AA’s high reactivity. The performance of ~50-nm AA-AuNPs was unstable when applied at various Ag concentration tests (fig. S26). Further improvement in synthesis control will probably increase the sensitivity of AA-AuNP LFIAs. Currently, the used ~30-nm AA-AuNPs are more stable and already perform better than the same-sized traditional Cit-AuNPs. The reliability of AA-AuNP LFIA was further ensured through recovery tests of different amounts of spiking (recovery rates, 84.2 to 110.7%; CV < 5%; table S4).

**Fig. 5.**
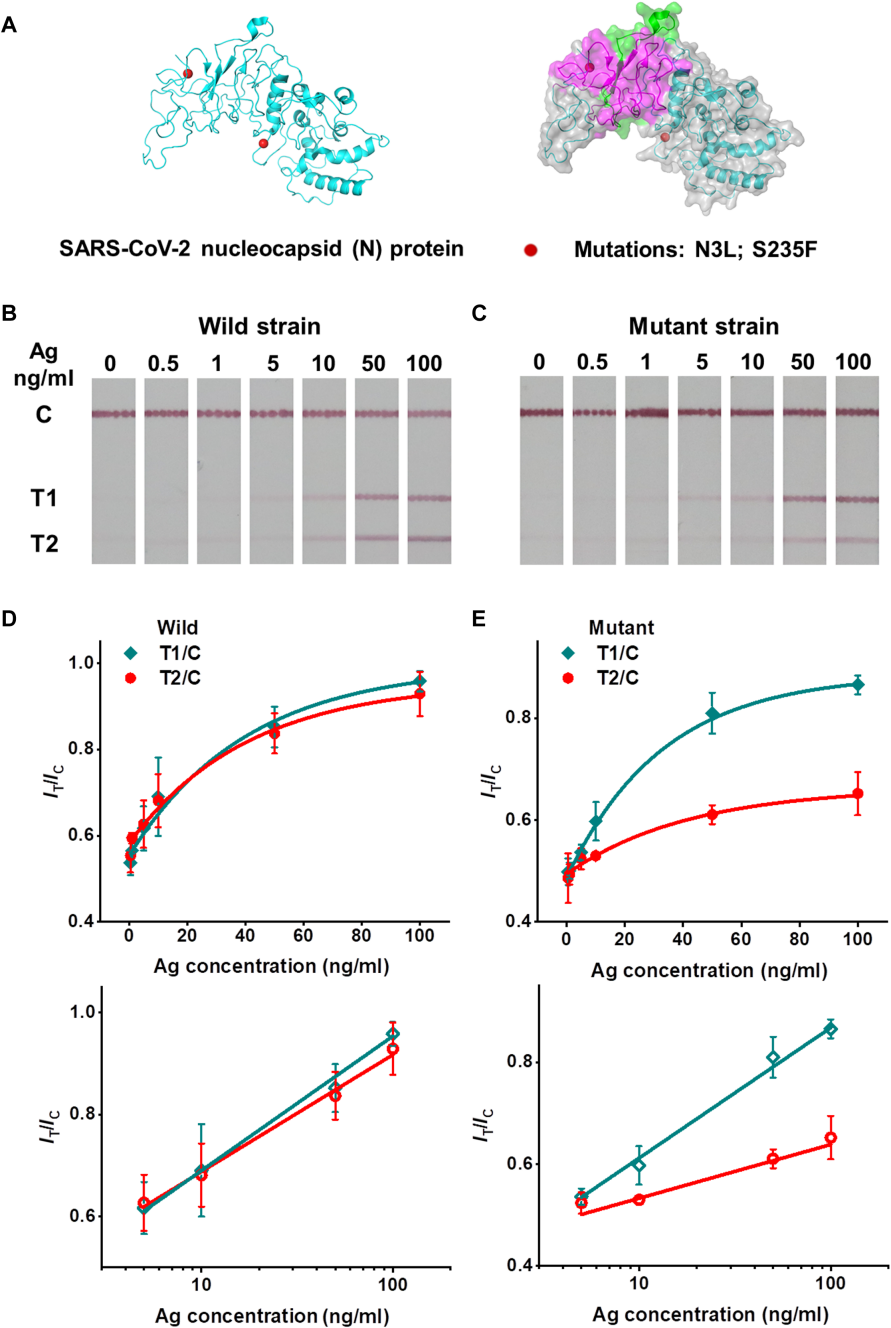
Virus mutant detection using AA-AuNP LFIAs. (**A**) The cartoon image of SARS-CoV-2 nucleocapsid (N) protein with mutation sites (red balls) of B.1.1.7 lineage. Right: The correlated surface covered the image of B.1.1.7 lineage N protein. The magenta surface represents the Ag site of the conformational epitope of N protein (44 to 175 amino acids) for test line T1. The green surface represents the Ag site of a linear epitope of N protein (74 to 105 amino acids) for control line C. (**B** and **C**) The testing strips of AA-AuNP LFIAs responding to different concentrations of wild strain N protein and the mutant B.1.1.7 lineage N protein. (**D** and **E**) The correlated concentration-dependent intensity ratio (*I*_T_/*I*_C_) curves (top) and linear fitted lines (bottom). The error bars represent three different replicates.

On the basis of the surface chemistry–mediated sensitization, we applied the weakly ionized AA-AuNP LFIA for virus mutant detection. Viruses are prone to mutation. Instant detection of mutant-infected individuals helps avoid the crowd-gathering risk and prevent the new epidemic outbreak. Recently, the SARS-CoV-2 pandemic caused a great disaster to global society. In a short time, SARS-CoV-2 developed many mutations and produced new epidemic mutant lineages, for example, the B.1.1.7 lineage. The earliest B.1.1.7 mutant strain has two mutation sites in the nucleocapsid (N) protein (D3L and S235F; Fig. 5A). We fabricated the AA-AuNP LFIA with dual test lines (T1 and T2) to recognize the wild and mutant strains. The label Ab and capture Ab_T1_ recognize a linear epitope Ag site (74 to 105 amino acids) and a conformational epitope Ag site (44 to 175 amino acids) of N protein, respectively. These two Ag sites are not involved with the mutations. The T1 line responds to the wild strain and the mutant strain. The capture Ab_T2_ recognizes the Ag site of wild strain N protein directly affected by the mutations. The mutation would weaken the specific binding between the capture Ab_T2_ and the Ag site. For the wild strain detection, T1 and T2 lines showed strong color signals (Fig. 5B). For the mutant strain, the T2 line color signal was weaker than the T1 line (Fig. 5C). Comparing the intensity ratio signal of *I*_T_/*I*_C_, the T2 line signal is nearly equal to the T1 line signal for the wild strain (Fig. 5D). However, because of the mutation-weakened binding affinity of Ab-Ag, the T2 line signal decreases markedly in contrast to the T1 line signal for the mutant strain (Fig. 5E). At the same concentrations, the mutant strain’s intensity ratio signal of *I*_T2_/*I*_T1_ is lower than the wild strain (fig. S27). By visually and quantitatively counting the T2 and T1 line signals, AA-AuNP LFIA successfully detects the wild virus and the variant bearing certain mutations. Owing to the convenience in the fabrication and operation, the ultrasensitivity, and the detection reliability, we are convinced of the advantages of weakly ionized AA-AuNP LFIAs as a promising and powerful candidate for a new generation of LFIAs.

## DISCUSSION

In summary, this study presented a surface chemistry strategy using weakly ionized AuNPs to sensitize immunoassays. We explored the proof-of-concept AA-AuNP LFIAs for detecting various serum biomarkers with an LOD in the pg/ml range. AuNP LFIAs are considered highly practical and powerful POC sensors, playing an essential role in different health and safety fields. The urgent need for improved detection sensitivity prompted the development of FL/CL/SERS/nanozyme LFIAs. Unlike these LFIAs that require complicated fabrications, auxiliary devices, and additional operations/reagents, we developed the weakly ionized AA-AuNP LFIAs by modulating the surface ligand of AuNPs from strongly ionized Cit to weakly ionized AA. The weakly ionized AA-AuNP LFIA exhibits hundreds of times higher sensitivity than the traditional strongly ionized Cit-AuNP LFIA. The straightforward AA-AuNP LFIA is either superior or equivalent to the complicated FL/CL/SERS/nanozyme LFIAs. On the basis of experimental tests and MD simulations, we investigated surface chemistry–mediated nanointerfacial interactions and clarified the sensitization mechanisms behind Ab@AA-AuNP complex: more Abs adsorption and higher bioactivity of adsorbed Abs. By combining MD simulation analysis with machine learning techniques, the weakly ionized AuNP concept will extend to screen other ligand-coated AuNPs to design ultrasensitive POC sensors. The surface chemical approach of weakly ionized AuNPs for sensitizing immunoassays not only is suitable for POC paper-based sensors but also has the potential to be applied in the development of high-throughput and multianalyte microfluidic chip sensors and flow cytometric immunoassays.

## MATERIALS AND METHODS

### Materials

HAuCl_4_ was from Sinopharm Chemical Reagent Co. Ltd. (Shanghai, China). AA sodium salt, Triton X-100, Tween 20, sodium chloride, saccharose, trisodium phosphate, and bovine serum albumin (BSA) were from Sigma-Aldrich (USA). Boric acid buffer (0.5 M), 10× phosphate buffer saline (PBS), PEG-4000, potassium peroxodisulfate, and potassium carbonate were from Macklin (Shanghai, China). SDS solution (10%), *N,N,N*′*,N*′-tetramethylethylenediamine, 30% acrylamide-bisacrylamide (29:1) solution, 1 M tris-HCl (pH 6.8), 1.5 M tris-HCl (pH 8.8), 10× tris-Gly running buffer, and Coomassie Blue fast staining solution and destaining solutions were from Beyotime Biotechnology (Beijing, China). Nitrocellulose (NC) membrane, glass fiber pad, absorbent pad, and polyvinyl chloride sheet were from Shanghai Jiening Biological Technology Co. Ltd. (Shanghai, China). Sodium Cit-AuNPs (30 nm) were from Nanjing Nanoeast Biological Technology (Nanjing, China). Goat anti-mouse IgG(H + L) (anti-Ab) was from ImmunoWay Biotechnology (USA). Fetal bovine serum was from Gibco (Australia). PCT 16A1 (capture Ab) was from Nanjing GenScript Biotechnology Co. Ltd. (Nanjing, China). AFP (Ag), AFP-28 (label Ab), AFP-25 (capture Ab), CRP (Ag), CRP-Ab9 (label Ab), CRP-Ab8 (capture Ab), PCT (Ag), PCT-Ab7 (label Ab), COV19-PS-MAb117 (label Ab), COV19-PS-Mab30/COV19-PS-MAb81 (T1/T2, capture Ab), and SARS-CoV-2 Ag (nucleocapsid protein, wild strain: ncov-PS-Ag19; B.1.1.7 mutant strain: ncov-PS-Ag40) were from Fapon Biotech (Dongguan, China). CRP nanobody (SAA1358) was from AntibodySystem (Schiltigheim, France).

### Instruments and characterizations

Transmission electron microscopy characterizations were performed using an HT7700 instrument (Hitachi, Japan). Samples were prepared by drop-casting on the carbon-coated copper grid. Ultraviolet (UV)–visible optical spectra were recorded using a UV-2600i spectrophotometer (Shimadzu, Japan). Dynamic light scattering hydrodynamic diameter profiles and zeta-potential distribution profiles were measured by a Zetasizer Nano ZS instrument (Malvern, UK). XPS characterizations are performed using an ESCALAB 250Xi instrument (Thermo Fisher Scientific, USA). The SDS-PAGE experiment was performed using the Bio-Rad electrophoresis system and analyzed by the ChemiDoc MP gel imager instrument (Bio-Rad, USA). A scribing instrument HGS510 (Fapon Biotech, China) was used to dispense the capture Ab and anti-Ab on the NC membrane. The intensity signals of the T and C lines were measured using a portable strip reader FIC-Q1 (Hangzhou Autokun Technology, China).

### Synthesis of AA-AuNPs

The weakly ionized AA-AuNPs were synthesized following our reported protocol with necessary modifications (*47*). All glass containers are cleaned using the aqua regia solution. (Cautions: Aqua regia solution is dangerous and should be operated after careful training and wearing protective equipment.) AA is used as the reducing and stabilizing agent to synthesize the AA-AuNPs with an approximate size of 30 nm. Deionized water (50 ml) containing HAuCl_4_ (1 mM) was added into a 100-ml round-bottom flask. After heating to 90°C, 0.1 ml of 1 M HCl solution and 1.5 ml of 1% AA solution were rapidly added under vigorous stirring for 20 min. The deep-red solution was cooled at room temperature and then centrifuged (8000 rpm, 10 min, and 4°C) to remove residual reagents.

### Synthesis of Ab-labeled AA-AuNP

Typically, the Ab-labeled AA-AuNP (Ab@AA-AuNP) for LFIAs was prepared by directly mixing AA-AuNPs with the label Ab (final concentration of 10 μg/ml). The mixture was placed on the shaker and incubated for 1 hour in the dark. Then, the mixture was added with 0.1% BSA to block the surface of AA-AuNP for a further 0.5-hour incubation. After centrifuging (8000 rpm, 10 min, and 4°C), the obtained Ab@AA-AuNP was concentrated and resuspended using 20 mM Na_3_PO_4_ buffer containing 5% BSA, 0.25% Tween 20, and 10% saccharose, stored at 4°C for usage.

When applied for UV-visible, TEM, XPS, dynamic light scattering, and SDS-PAGE analysis, the operations were the same except for the BSA-blocking treatment.

### Synthesis of Ab@Cit-AuNP

To prepare the Ab@Cit-AuNP, the pH of Cit-AuNPs solution was altered to 8.5 by adding K_2_CO_3_ to avoid aggregation when incubated with Abs. Then, Cit-AuNPs were added with the label Ab (final concentration of 10 μg/ml), placed on the shaker, and incubated for 1 hour in the dark. The surface of Cit-AuNP was blocked using 0.1% BSA for a further 0.5-hour incubation. After centrifuging (8000 rpm, 10 min, and 4°C), the obtained Ab@Cit-AuNP was concentrated and resuspended using 20 mM Na_3_PO_4_ buffer containing 5% BSA, 0.25% Tween 20, and 10% saccharose, stored at 4°C for usage. When applied for characterization and chemical analysis, the operations were the same except for the BSA-blocking treatment.

### Fabrication of the AuNP LFIAs

The test strips were fabricated following the reported protocols. Typically, the strip consists of the sample pad, conjugate pad, NC chromatography membrane, and absorbent pad attached to the polyvinyl chloride sheet. The sample pad (glass fiber) was soaked in the boric acid buffer (0.2 M, pH 9) containing 2% Triton X-100, 1.8% NaCl, 1% BSA, and 2% PEG-4000 and dried at 37°C for usage. The conjugate pad (glass fiber) was used without any treatment. For the test of AFP, CRP, and PCT, we used a scribing machine to dispense the capture Ab (1 mg/ml) and anti-Ab (0.5 mg/ml) onto the NC membrane at a rate of 0.3 μl/cm to form a test line (T) and a control line (C), respectively. For the test of SARS-CoV-2 mutant, we used a scribing machine to dispense the capture Ab_T1_/Ab_T2_ (0.9 mg/ml) and anti-Ab (1 mg/ml) onto the NC membrane at a rate of 0.3 μl/cm to form a test line (T) and a control line (C), respectively. The dispensed NC membrane was dried at 37°C overnight for usage. We used a paper-cutting machine to cut the assembled sheet materials into 3-mm-wide strips. At last, 1 μl/1.5 μl/1 μl/2 μl concentrated Ab_AFP_/Ab_CRP_/Ab_PCT_/Ab_SARS-CoV-2_-labeled AuNPs were dropped on the conjugate pad. The strips were dried at 37°C and sealed for usage.

### Sensitivity tests of the AA-AuNP LFIAs

We used three serodiagnostic biomarkers of AFP, CRP, and PCT to assess the sensitivity of the designed weakly ionized AA-AuNP LFIAs. A series of dilutions of these biomarkers were prepared using the running buffer (PBS, containing 1% Triton X-100). The sample solution (40 μl) was dropped on the sample pad. After 15 min, we measured the intensity signal of the T and C lines using the portable strip reader FIC-Q1. The concentration-dependent intensity ratios of T/C (*I*_T_/*I*_C_) were plotted and analyzed with linear fitting to calculate the LOD value and the quantitative detection range. For the test of SARS-CoV-2 mutant, 70 μl of sample solution was dropped on the sample pad.

### Clinical samples

The clinical serum samples were collected from the Department of Hepatobiliary and Pancreas Surgery, Shenzhen People’s Hospital (The First Affiliated Hospital of Southern University of Science and Technology) and approved by the institutional ethics committee (LL-KT-2018207).

### Clinical sample tests using AA-AuNP LFIAs

We used AA-AuNP LFIAs to detect AFP in clinical serum samples. The serum samples were mixed with 1% Triton X-100 solution at a volume ratio 1:1. Then, we added 40 μl of sample solution to the sample pad. After 15 min, we imaged and measured the color intensity of the T and C lines of the strips. A linear equation regressed the intensity ratio of T/C (*I*_T_/*I*_C_) to calculate the concentration of AFP. We assessed the reliability of AA-AuNP LFIAs by analyzing the correlation to the clinic method (CLIA).

### MD simulation

MD simulations were performed for an Au(111) surface loaded with AA^−^/Cit^3−^ and IgG protein by GROMACS 2021.5 package (*62*). The initial structure of the Au(111) surface (20.1 nm by 20.4 nm) containing 34,440 Au atoms was obtained from the CHARMM-GUI website (*63*). Cit^3−^ and ascorbate ion AA^−^ were geometrically optimized by Gaussian 16 A.03 under density functional theory B3LYP/def-TZVP level with DFT-D3 dispersion correction and SMD implicit solvent model (*64*). AmberTools21 and ACPYPE were used to construct the general AMBER force field 2 (GAFF2) parameters (*65*–*67*). Multiwfn was used to fit the restrained electrostatic potential 2(RESP2) charge (*68*, *69*). Protonation models of IgG at pH 8.5 and pH 7 were established based on PROPKA 3.1-predicted p*K_a_* and were parameterized by an Amberff14sb force field (*70*, *71*).

A box (20.1 nm by 20.4 nm by 19.2 nm) was built with a prebalanced Au surface and Cit^3−^/AA^−^ systems with different protonation states of IgG (pH 8.5 for Cit^3−^-loaded Au surface and pH 7 for AA^−^-loaded Au surface). Different protonated IgG was placed above the Au surface. For both systems, 798 Cit^3−^ or 700 AA^−^ were initially absorbed on the Au surface. Systems were solvated in TIP3P water, and a necessary amount of Na^+^ was added to keep them electrically neutral. Energy minimization was performed using the steepest descent algorithm with a force tolerance of 500 kJ mol^−1^ nm^−1^. In all directions, periodic boundary conditions were imposed. Systems were annealed for 1 ns under NVT ensemble from 0 to 300 K and relaxed for 1 ns under NPT ensemble, which position restraints with a constant of 1000 kJ mol^−1^ nm^−2^ in three directions that were performed on Au atoms, Cit^3−^/AA^−^ ions, and IgG.

After completing the above steps, 100-ns NPT MD simulations were performed on Au@Cit^3−^/Au@AA^−^ and IgG systems with position restraints on Au atoms. The pressure was maintained at 1 bar by the Parrinello-Rahman barostat in a semi-isotropic manner (*xy* and *z* directions), and the temperature was maintained at 300 K by the V-rescale thermostat (*72*, *73*). The LINCS algorithm was performed to constrain the bond lengths of hydrogen atoms. Lennard-Jones interactions were calculated within a cutoff of 1.2 nm, and electrostatic interactions beyond 1.2 nm were treated with the particle-mesh Ewald (PME) method with a grid spacing of 0.16 nm. UCSF ChimeraX was used to visualize simulation results (*74*).
