# Supplementary material for: Weakly ionized gold nanoparticles amplify immunoassays for ultrasensitive point-of-care sensors
Source: Sci Adv. 2024 Jul 10;10(28):eadn5698. doi: 10.1126/sciadv.adn5698 (PMC11235179; doi:10.1126/sciadv.adn5698)
Supplement: Supplementary file 1 — Figs. S1 to S27 Tables S1 to S4 Legends for movies S1 and S2 [file sciadv.adn5698_sm.pdf]

Science Advances
AAAS

Supplementary Materials for

Weakly ionized gold nanoparticles amplify immunoassays for ultrasensitive point-of-care sensors

Jiangjiang Zhang *et al.*

Corresponding author: Xingyu Jiang, jiang@sustech.edu.cn; Jiangjiang Zhang, zhangjj@bit.edu.cn;
Dou Wang, wangd9@sustech.edu.cn

Sci. Adv. **10**, eadn5698 (2024)
DOI: 10.1126/sciadv.adn5698

The PDF file includes:

Figs. S1 to S27
Tables S1 to S4
Legends for movies S1 and S2

Other Supplementary Material for this manuscript includes the following:

Movies S1 and S2

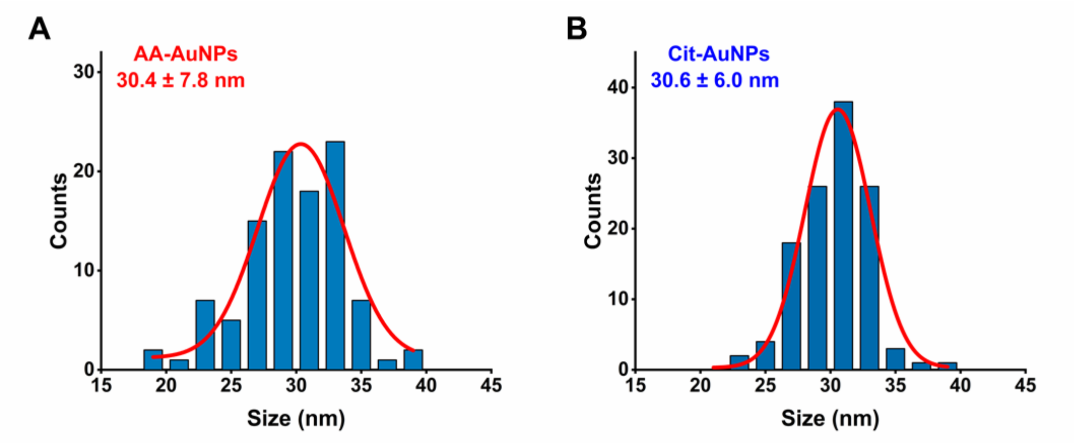

Fig. S1. Characterizations of AA-AuNPs and Cit-AuNPs. The size distribution profiles of the weakly ionized AA-AuNPs (A) and the strongly ionized Cit-AuNPs (B), counting by the TEM measurements (each over one hundred particles).

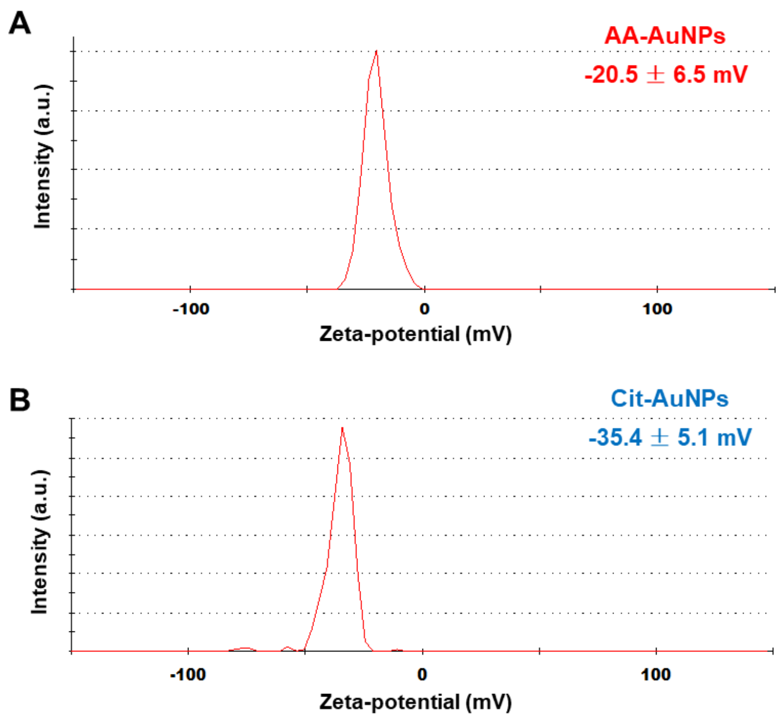

Fig. S2. Characterizations of AA-AuNPs and Cit-AuNPs. The zeta-potential distribution profiles of the weakly ionized AA-AuNPs (A) and the strongly ionized Cit-AuNPs (B).

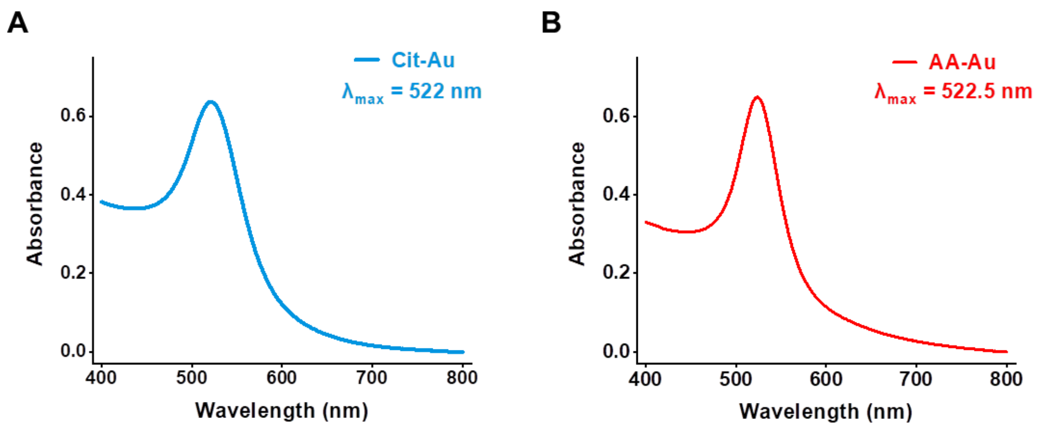

Fig. S3. Characterizations of AA-AuNPs and Cit-AuNPs. The absorption spectra of the strongly ionized Cit-AuNPs (A) and the weakly ionized AA-AuNPs (B).

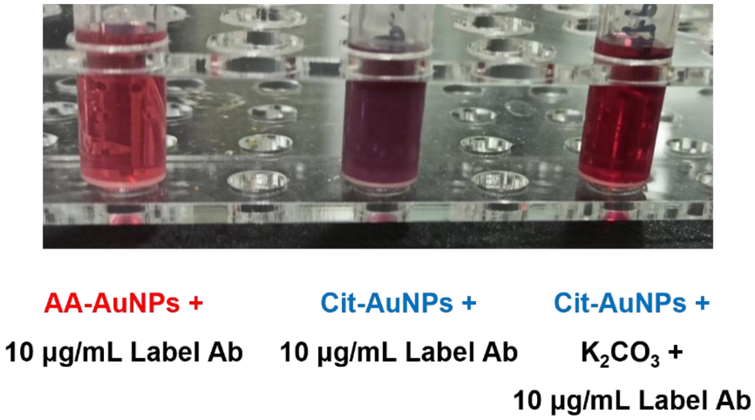

Fig. S4. Characterizations of AA-AuNPs and Cit-AuNPs for preparing Ab@AuNPs complex. The photograph of the strongly ionized Cit-AuNPs and the weakly ionized AA-AuNPs during incubation with the same amount of Ab.

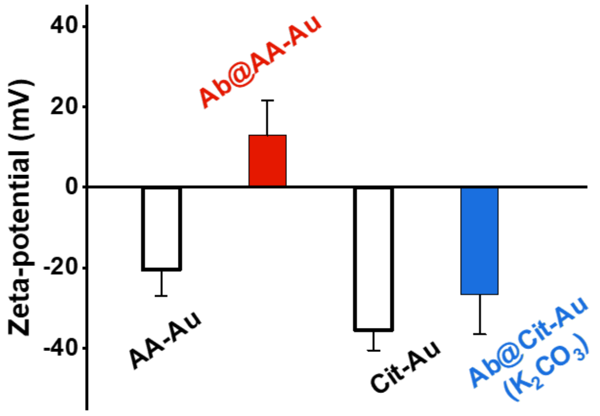

Fig. S5. Characterizations of AA-AuNPs, Cit-AuNPs, and the relevant Ab@AuNPs complex. The zeta potential values of the strongly ionized Cit-AuNPs, the weakly ionized AA-AuNPs, the colloidal Ab@Cit-AuNP complex, and the colloidal Ab@AA-AuNP complex.

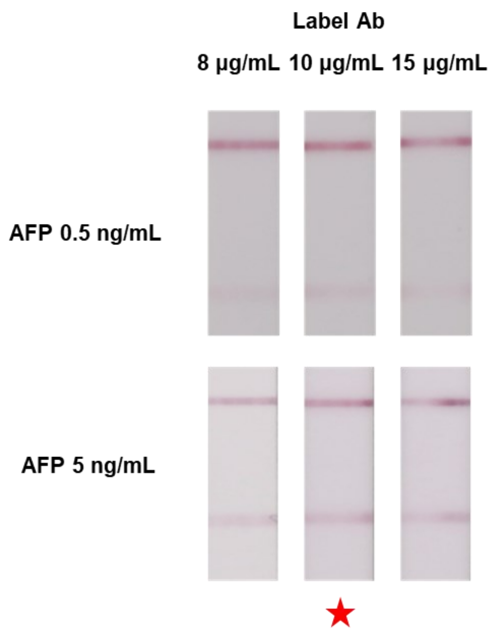

Fig. S6. Optizations of AA-AuNPs LFIA for AFP. The photographs of tested strips of the weakly ionized AA-AuNPs LFIAs responding to different concentrations of AFP when the label Ab concentrations are 8 μg/mL, 10 μg/mL, and 15 μg/mL, respectively.

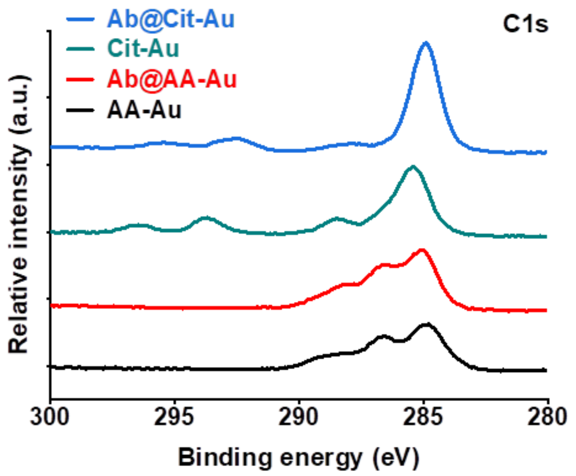

Fig. S7. Characterizations of AA-AuNPs, Cit-AuNPs, and the relevant Ab@AuNPs complex. The C1s XPS binding energy profiles of the strongly ionized Cit-AuNPs, the weakly ionized AA-AuNPs, the colloidal Ab@Cit-AuNP complex, and the colloidal Ab@AA-AuNP complex.

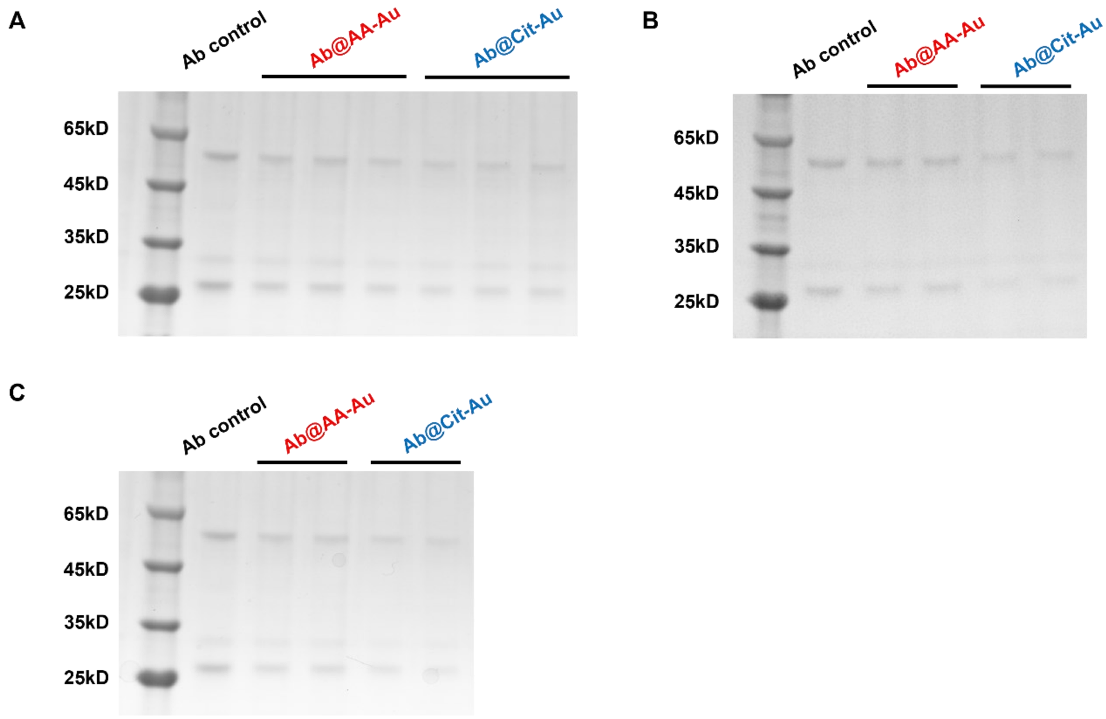

Fig. S8. Characterizations of Ab@AA-AuNPs and Ab@Cit-AuNPs complex. (A) ~ (C) The gel images of the detached protein from collected Ab@AA-AuNPs and Ab@Cit-AuNPs complexes after SDS-PAGE analysis (stained with Coomassie brilliant blue). Each gel contains the Ab control and duplications of Ab@AA-AuNPs and Ab@Cit-AuNPs groups.

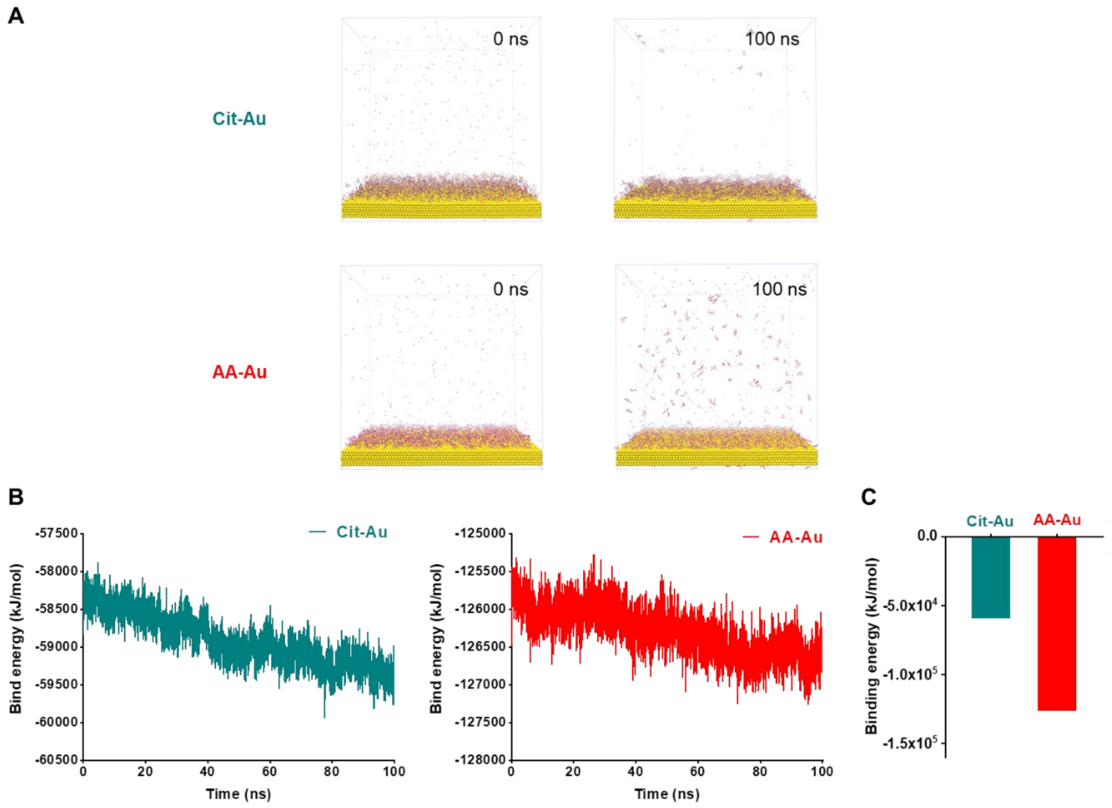

Fig. S9. Theoretical calculation studies of AA-Au and Cit-Au surfaces. (A) Molecules dynamic (MD) simulation snapshot pictures (0 ns and 100 ns) of Cit and AA on the surface of Au(111) (~ 20 × 20 nm^2). At the original state, 800 Cit^{3-} or AA^- molecules are loaded (density ~ 2/nm^2) under pH 8.5 or 7, respectively. The necessary amount of sodium ions was loaded to balance the charges. For a clear view, water molecules and non-polar H atoms are hidden. After 100 ns simulation, 19 Cit^{3-} or 100 AA^- diffused and detached from the Au surface. These molecules were removed in nest simulations. (B) The time-dependent total binding energy profiles of Au surface and Cit^{3-}/AA^- systems during the MD simulations. (C) The average binding energy intensities of Au surface and Cit^{3-}/AA^- systems at the last 10 ns simulations.

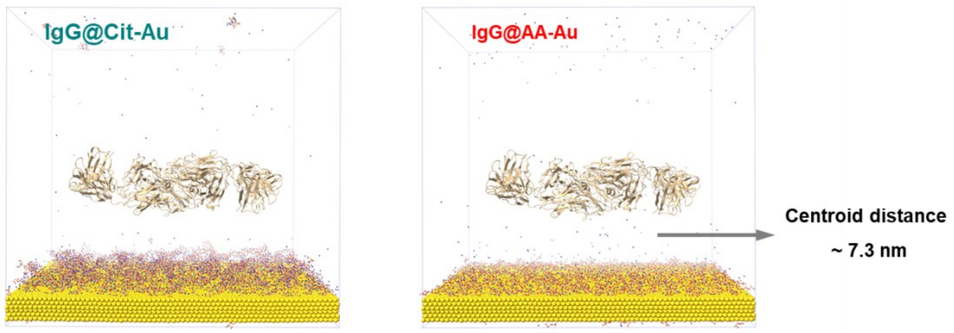

Fig. S10. Theoretical calculation studies of IgG@AA-Au and IgG@Cit-Au systems. The cartoon presentation of the initial states of the IgG@Cit-Au and IgG@AA-Au systems.

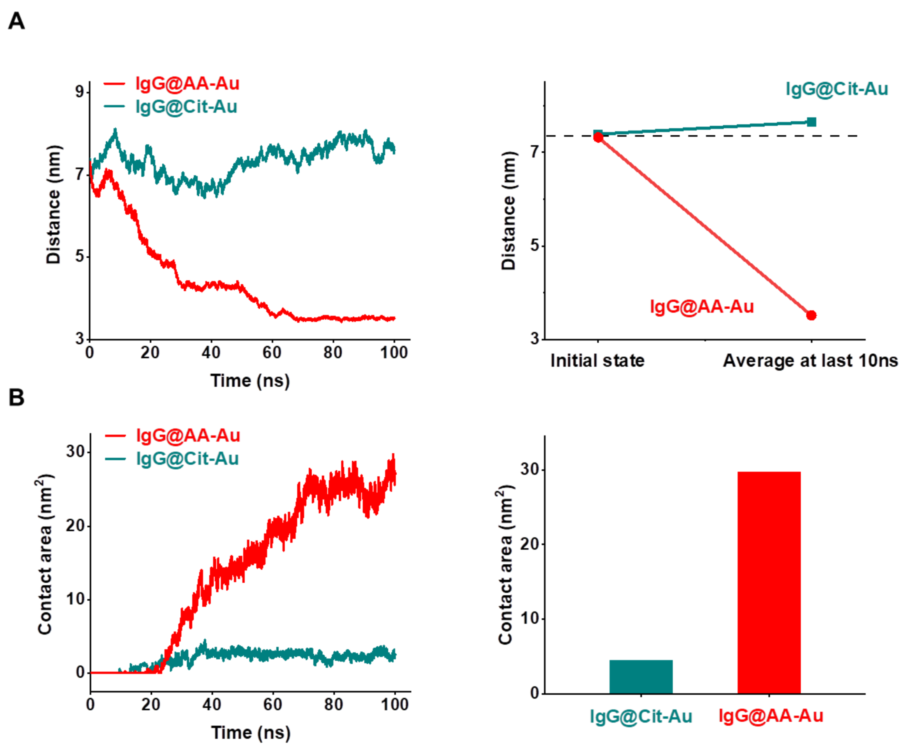

Fig. S11. MD studies of IgG@AA-Au and IgG@Cit-Au systems. (A) The time-related centroid distance curves between IgG molecule and Cit-Au surface/AA-Au surface during the MD simulation. Right: the average centroid distance change at the last 10 ns simulation. (B) The time-related contact area curves of IgG@Cit-Au system and IgG@AA-Au system during the MD simulation. Right: the average contact area at the last 10 ns simulation.

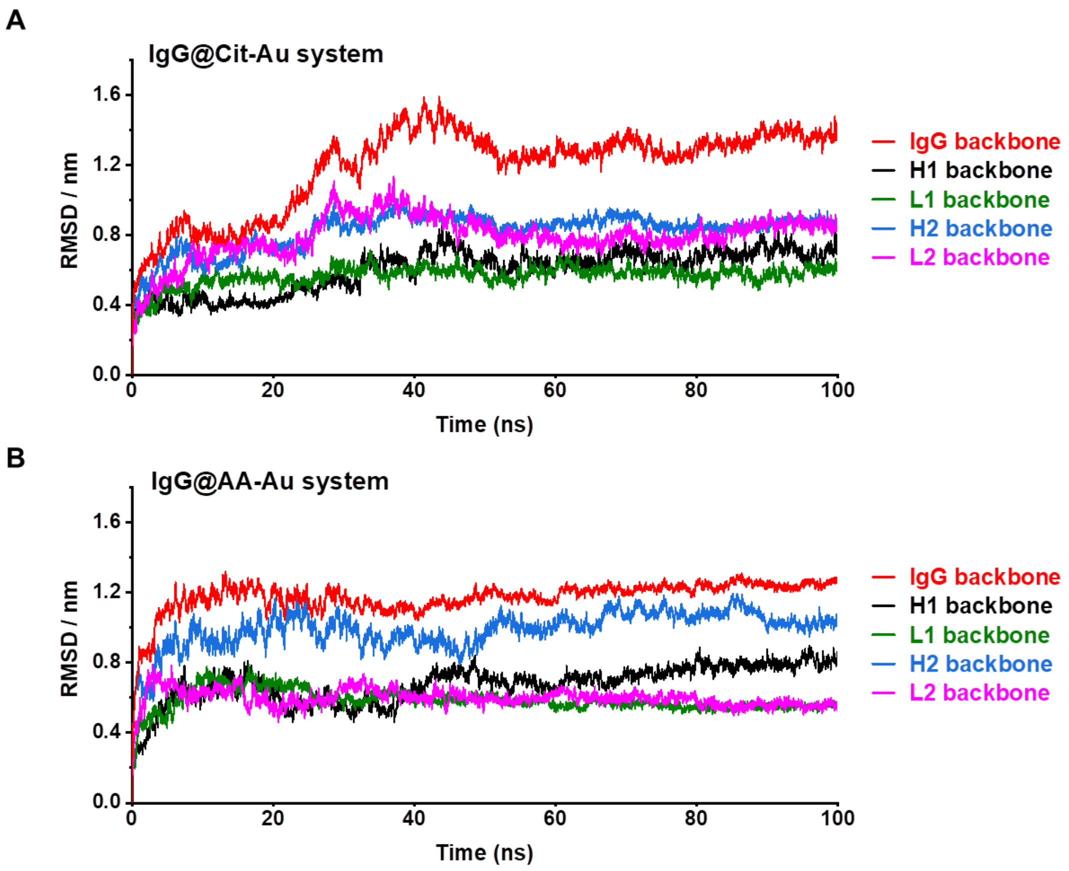

Fig. S12. MD studies of IgG@AA-Au and IgG@Cit-Au systems. The time-related root-mean-square error (RMSD) curves of the backbone atoms of IgG and different subunits versus their initial ground state for the IgG@Cit-Au system (A) and IgG@AA-Au system (B) during the MD simulation.

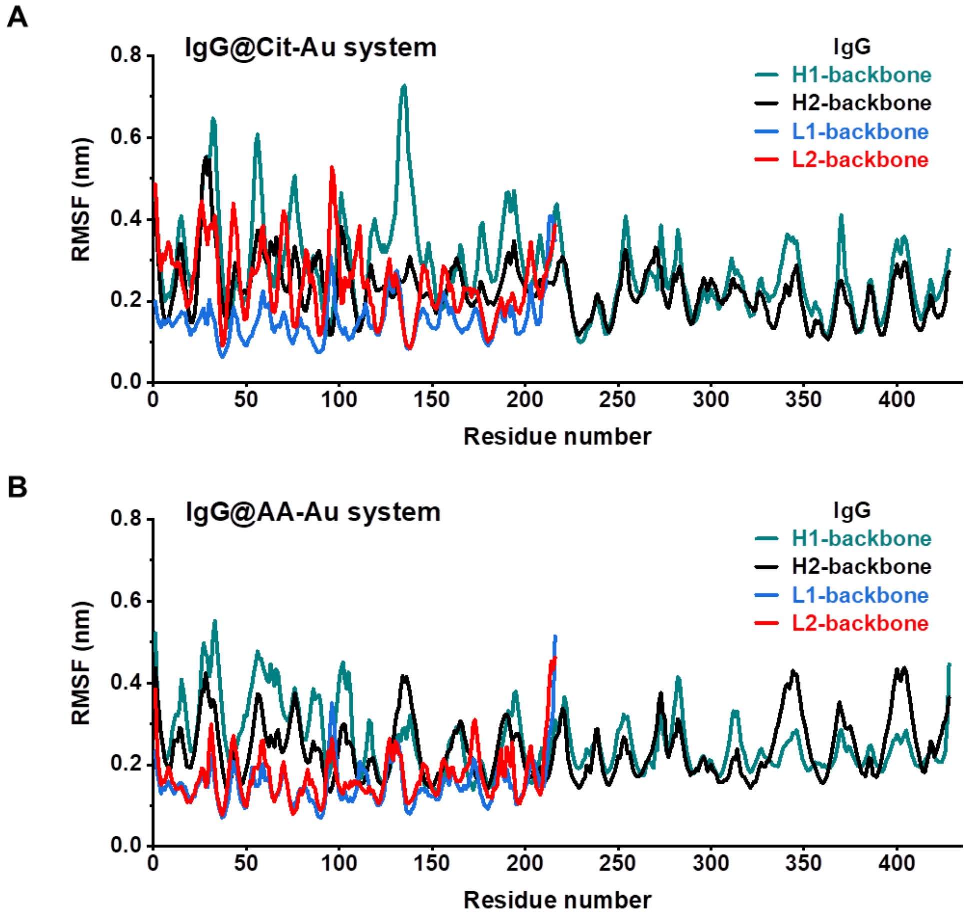

Fig. S13. MD studies of IgG@AA-Au and IgG@Cit-Au systems. The root mean square fluctuation (RMSF) curves of the residues' movement of different subunits of IgG for the IgG@Cit-Au system (A) and IgG@AA-Au system (B) during the MD simulation.

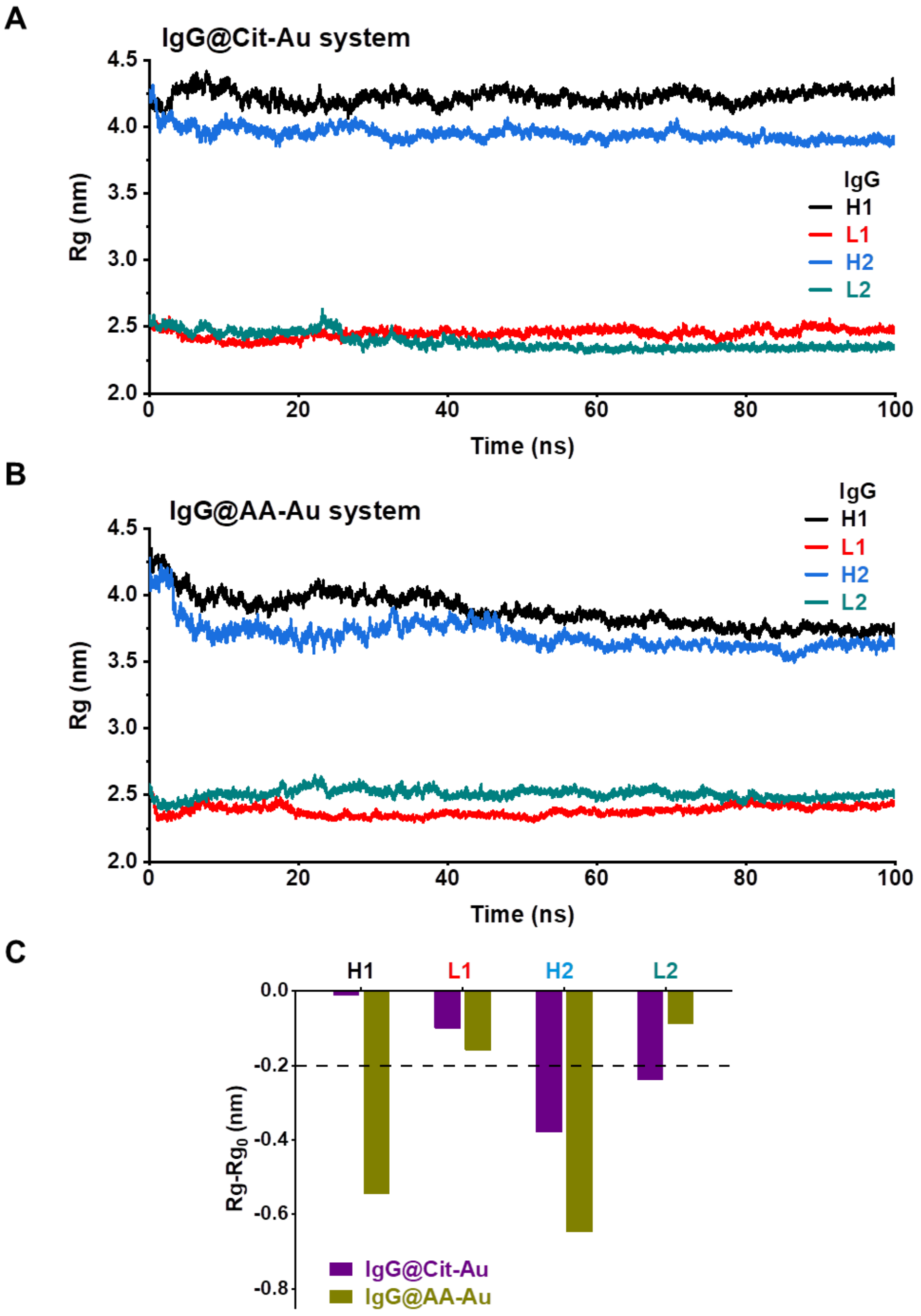

Fig. S14. MD studies of IgG@AA-Au and IgG@Cit-Au systems. The time-related gyration radius (Rg) curves of different subunits of IgG for the IgG@Cit-Au system (A) and IgG@AA-Au system (B) during the MD simulation. (C) The average Rg reduction of different subunits of IgG at the last 10 ns simulation.

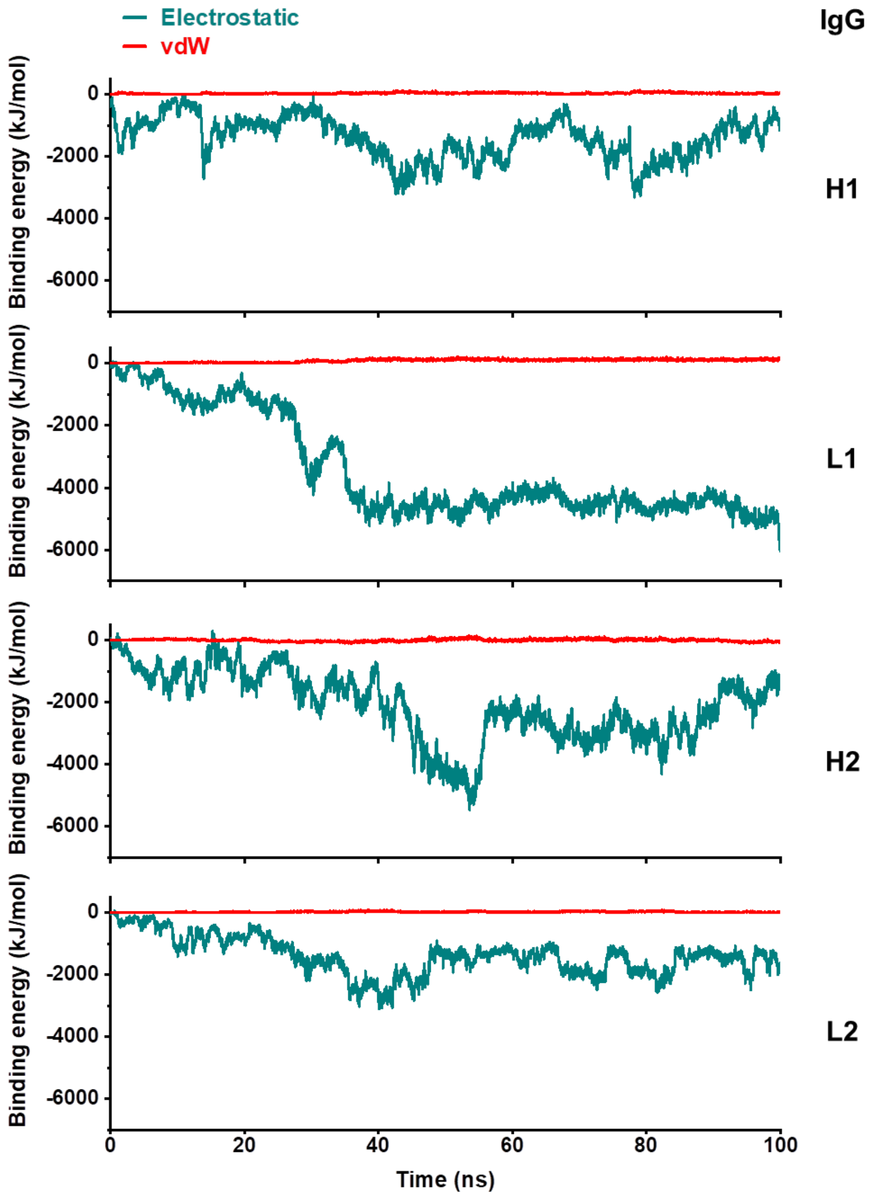

Fig. S15. MD study of IgG@Cit-Au system. The time-related energy curves of different subunits of IgG for the IgG@Cit-Au system during the MD simulation.

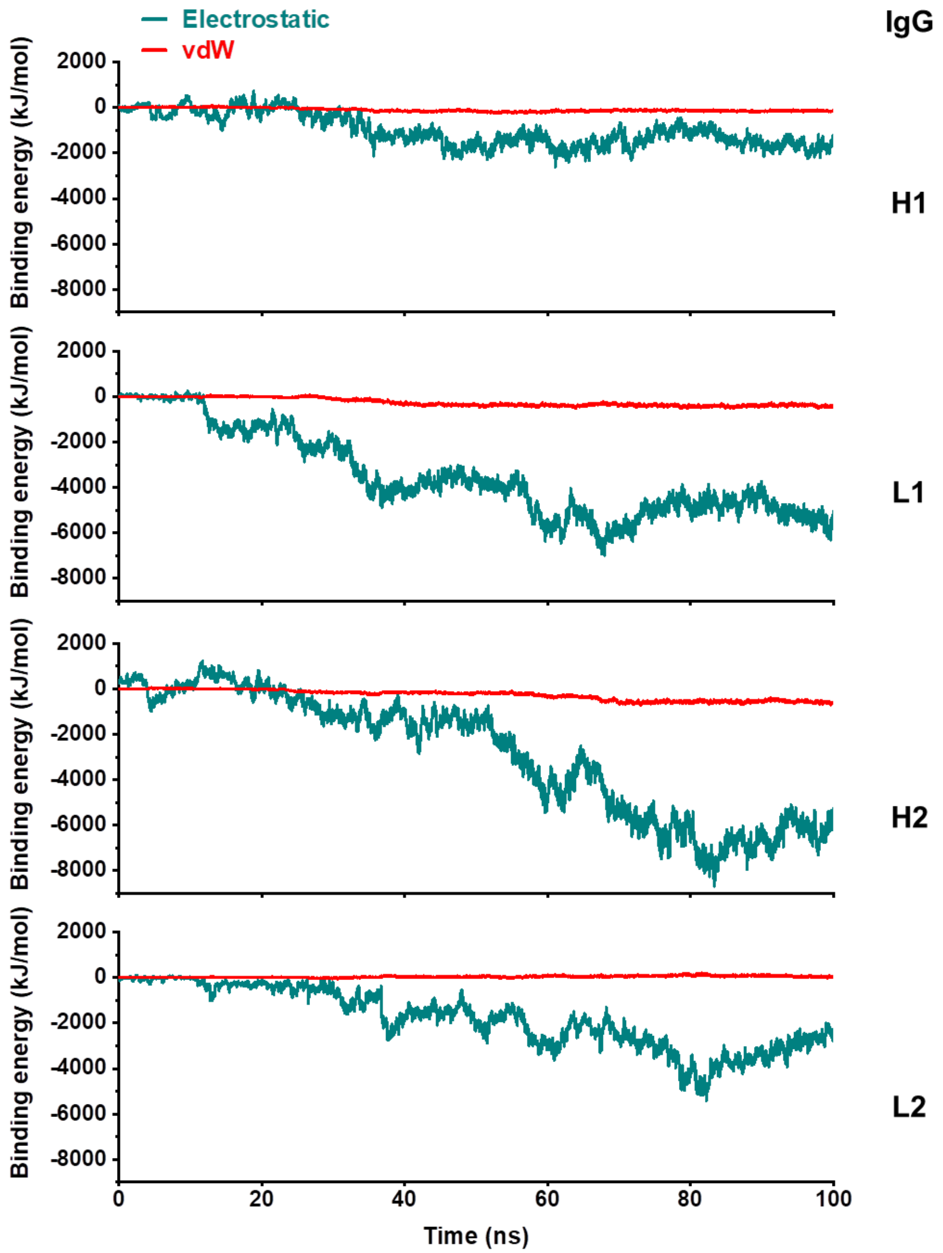

Fig. S16. MD study of IgG@AA-Au system. The time-related energy curves of different subunits of IgG for the IgG@AA-Au system during the MD simulation.

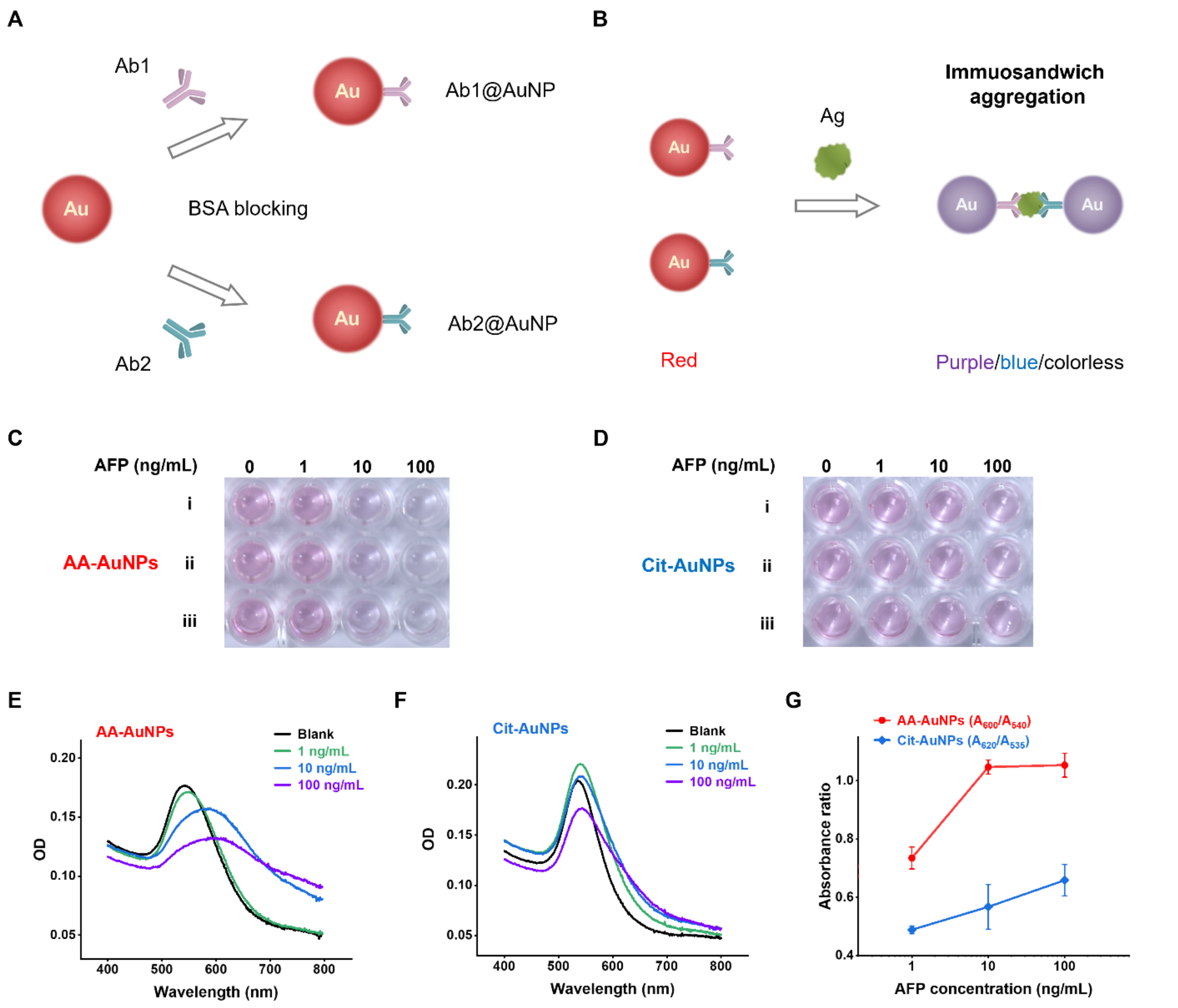

Fig. S17. Experimental studies of the binding interaction between the Ag and Ab@AuNPs. (A) Schematic illustration of the preparation of Ab1$_{AFP}$@AuNPs and Ab2$_{AFP}$@AuNPs. (B) Schematic illustration of the Ag$_{AFP}$-induced aggregation of Ab1$_{AFP}$@AuNPs and Ab2$_{AFP}$@AuNPs. (C) The photograph of the mixture of Ab1$_{AFP}$@AA-AuNPs and Ab2$_{AFP}$@AA-AuNPs responding to different concentrations of AFP (three duplications). (D) The photograph of the mixture of Ab1$_{AFP}$@Cit-AuNPs and Ab2$_{AFP}$@Cit-AuNPs responding to different concentrations of AFP (three duplications). (E) and (F) The corresponding absorption spectra. (G) The relevant absorbance ratio curves. The error bars represent three duplications.

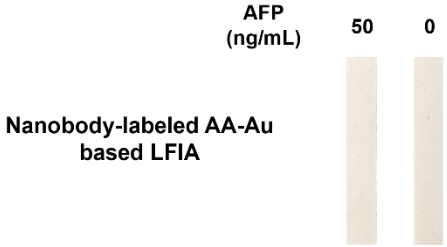

Fig. S18. Nanobody-based AA-AuNPs LFIA for CRP. The photographs of tested strips of nanobody$_{CRP}$-labeled AA-AuNPs based LFIA responding to different amounts of CRP.

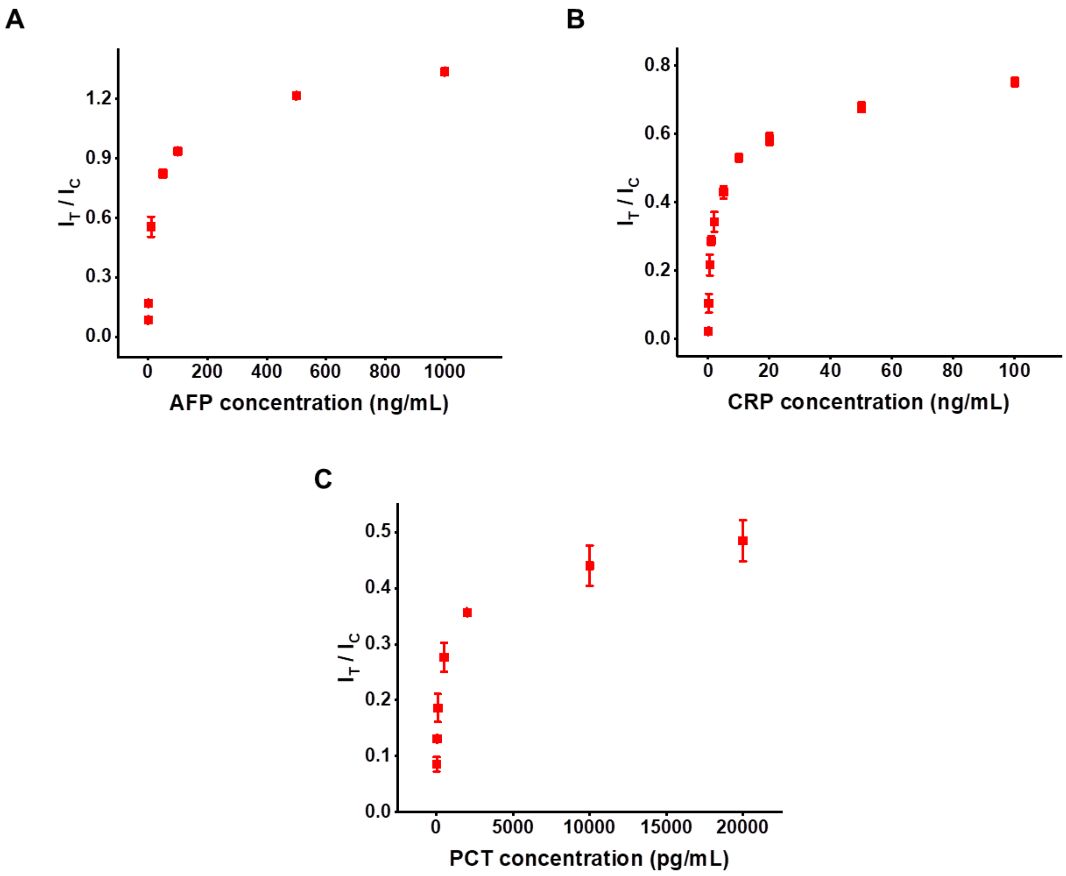

Fig. S19. AA-AuNPs LFIAs for different biomarkers. (A), (B), and (C) The concentration-dependent intensity ratio (I_T/I_C) curves of weakly ionized AA-AuNPs LFIAs responding to AFP, CRP, and PCT, respectively.

Table S1. The information of different LFIAs.

Methods	Materials	Marker	Detection range (pg/mL)	LOD (pg/mL)	Auxiliary	Cost efficiency	Ref.
**PL**	$NaYF_4$:Yb,Nd@$NaYF_4$	AFP	2000–200000	742	UV light or Laser	Low	(*25*)
	SiO_2@CdSe/CdS/ZnS	CRP	125–6250	90			(*22*)
	$NaYF_4$:Yb,Tm@$NaYF_4$	PCT	50–50000	30			(*53*)
**CL**	HRP@AuNPs	AFP	1000–200000	270	Reagents and Detector	Low	(*9*)
	HRP	CRP	100–100000	100			(*54*)
	ALP	PCT	44–100000	44			(*55*)
**C**	Cit-AuNPs	AFP	5000–200000	5000	/	High	(*9*)
		CRP	40000–2500000	5000			(*56*)
		PCT	490–14000	100			(*57*)
	AA-AuNPs	AFP<br>CRP<br>PCT	500–1000000<br>100–100000<br>20–20000	40<br>20<br>3	/	High	**This work**
**Nanoenzyme**	Au@PdNPs	CRP	500–100000	320	Reagents	Medium	(*58*)
	Fe3O4@MOF@PtNPs	PCT	5–140	0.5			(59)
**SERS**	DTNB-AuNRs	AFP	100–500000	10	Laser and Detector	Poor	(*39*)
	MBA-Au@SiO_2 NPs	CRP	500–1000000	50			(*60*)
	AuAg-ATP@Ag NPs	PCT	180–13000	30			(*61*)

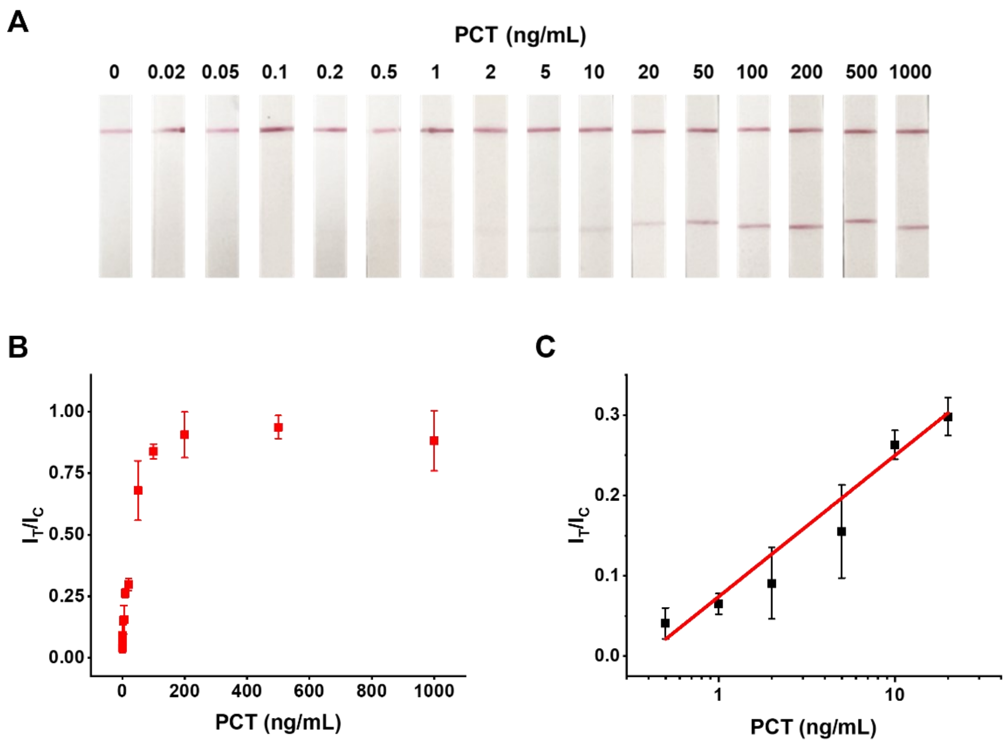

Fig. S20. Cit-AuNPs LFIA for PCT. (A) The testing strips of Cit-AuNPs LFIA responding to different concentrations of PCT at the optimal conditions in this work. (B) and (C) The corresponding concentration-dependent intensity ratio (I_T/I_C) and linear fitting curves (Adj.R^2=0.973).

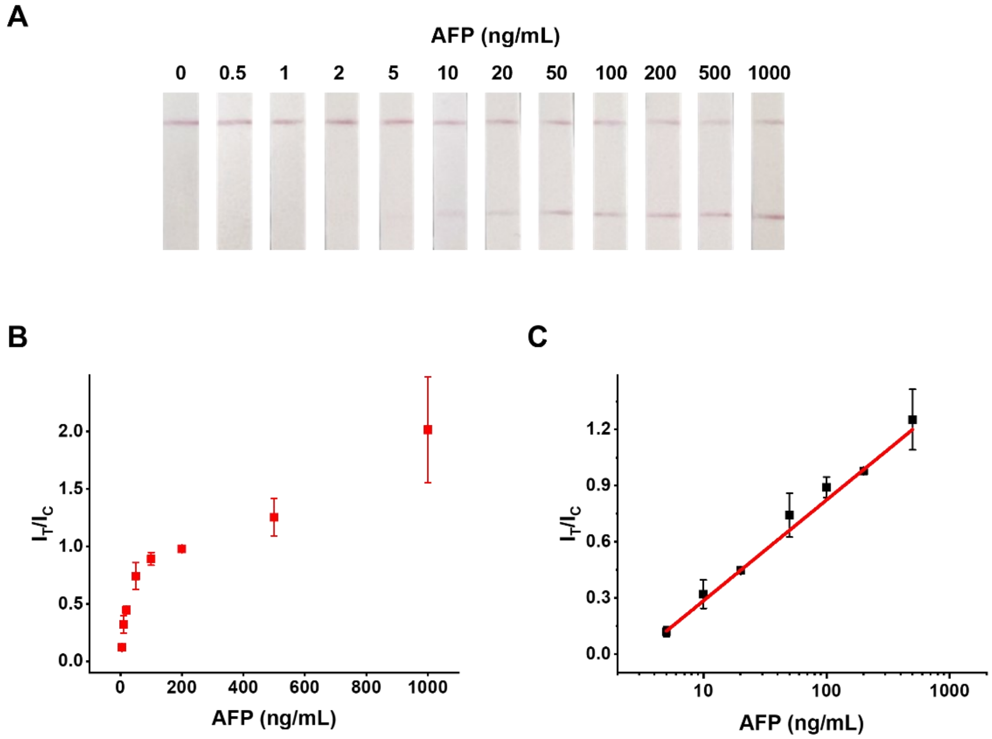

Fig. S21. Cit-AuNPs LFIA for AFP. (A) The testing strips of Cit-AuNPs LFIA responding to different concentrations of AFP at the optimal conditions in this work. (B) and (C) The corresponding concentration-dependent intensity ratio (I_T/I_C) and linear fitting curves (Adj.R^2=0.9971).

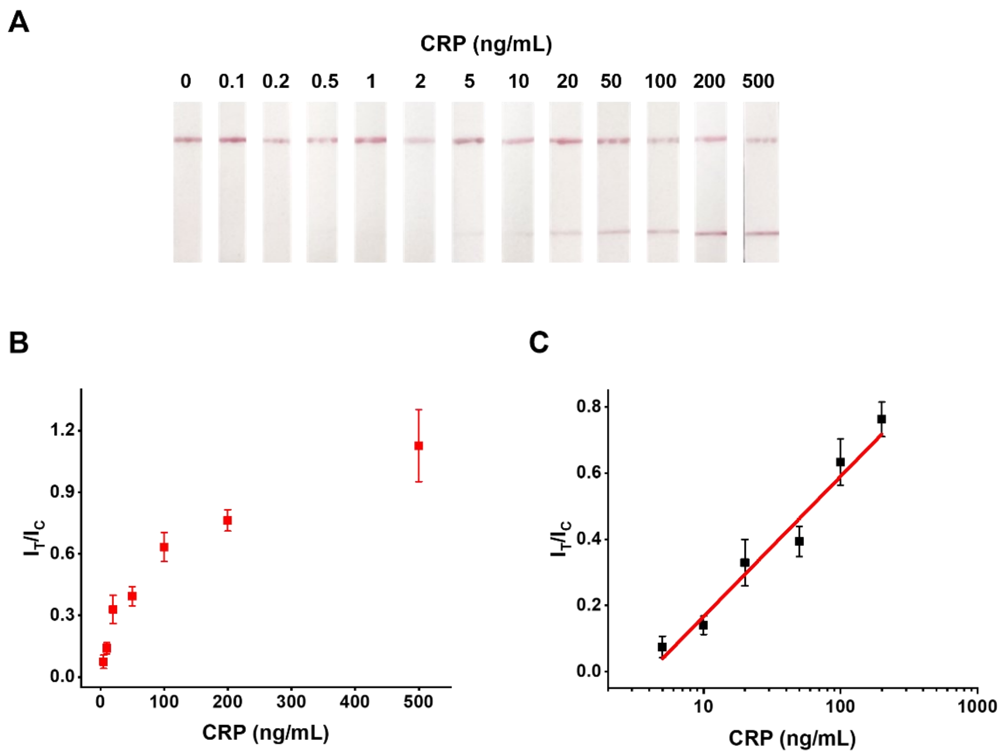

Fig. S22. Cit-AuNPs LFIA for CRP. (A) The testing strips of Cit-AuNPs LFIA responding to different concentrations of CRP at the optimal conditions in this work. (B) and (C) The corresponding concentration-dependent intensity ratio (I_T/I_C) and linear fitting curves (Adj.R^2=0.961).

Table S2. The comparisons between AA-AuNPs LFIA and Cit-AuNPs LFIA for AFP, CRP, and PCT detections under the optimal conditions in this work.

	AA-AuNPs LFIA		Cit-AuNPs LFIA		Sensitivity enhancement	
	Detection range	Visual[a] / LOD[b]	Detection range	Visual[a] / LOD[b]		
	ng/mL		ng/mL		visual[a]	LOD[b]
**AFP**	0.5 ~ 1000	0.5 / 0.04	5 ~ 500	10 / 0.15	20	3.75
**CRP**	0.1 ~100	1 / 0.02	5 ~ 200	10 / 0.23	10	11.5
**PCT**	0.02 ~ 20000	0.05 / 0.003	0.5 ~ 20	5 / 0.33	100	110

[a] The naked eye recognizable detection limit.
[b] The limit of detection calculated by 3σ/Slope.

Table S3. The information of the clinical samples.

Clinical ID	Sample ID	Age	Gender (Female/Male)	Clinical diagnosis
00941320	1	51	Female	Lithiasis
00930455	8	63	Female	Lithiasis
00932363	2	85	Male	Hepatapostema
00939473	10	66	Female	Lithiasis
00943198	3	63	Female	Lithiasis
00943503	7	68	Male	Lithiasis
00941164	9	29	Female	Hemangioma
00932559	6	54	Male	Lithiasis
00928561	5	33	Male	Lithiasis
00764722	4	78	Female	Papilloma (biliary)
00794789	15	51	Female	HCC (M)
00928255	16	47	Male	HCC (P)
00938512	18	33	Male	HCC (M-P)
00933703	19	46	Male	HCC (M-P)
00632475	13	62	Male	HCC (M)
00726721	17	63	Male	HCC (M-P)
00942595	11	81	Male	HCC (M)
00935885	12	46	Male	HCC (P)
00936911	14	33	Male	Metastasis HCC

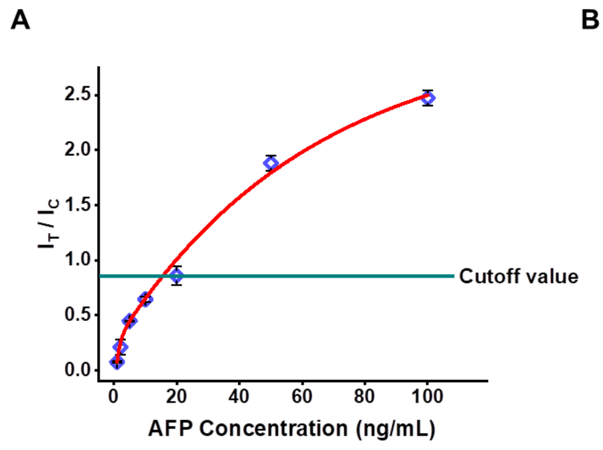

Fig. S23. AA-AuNPs LFIA for AFP in serum. The concentration-dependent curve (A) and the linear fitted line (B) of the intensity ratio (I_T/I_C) of the weakly ionized AA-AuNPs LFIAs responding to AFP in the spiked serum samples.

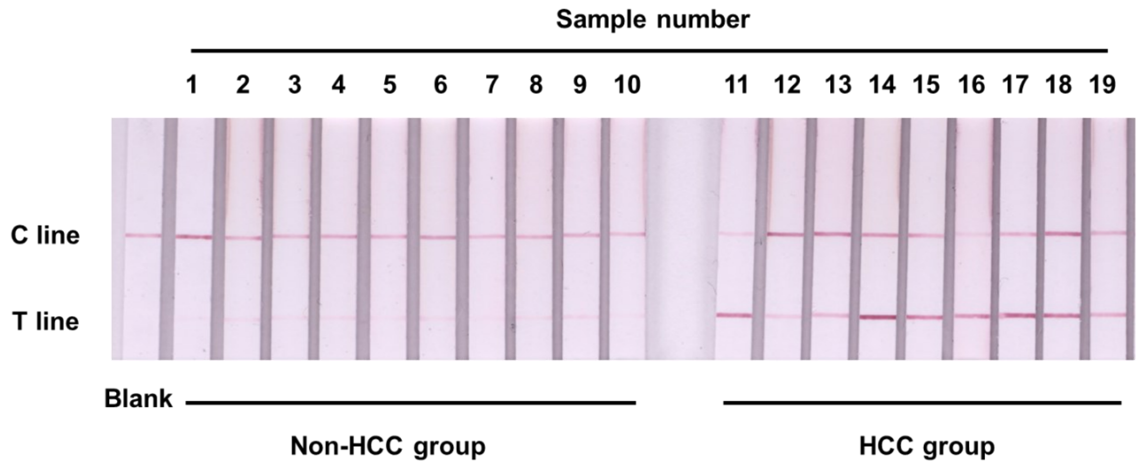

Fig. S24. AA-AuNPs LFIA for AFP in clinical samples. The photographs of tested strips of AA-AuNPs LFIAs responding to different clinical serum samples.

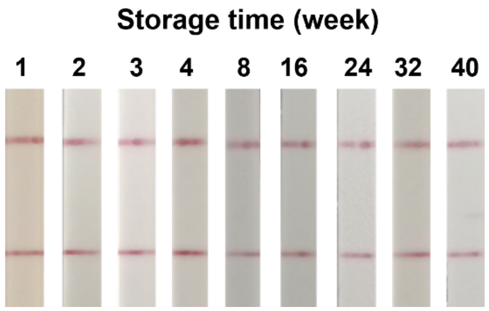

Fig. S25. Stability tests of AA-AuNPs LFIA. The photographs of the tested strips of AA-AuNPs LFIA after different storage times responding to 100 ng/mL AFP at the same conditions under room temperature.

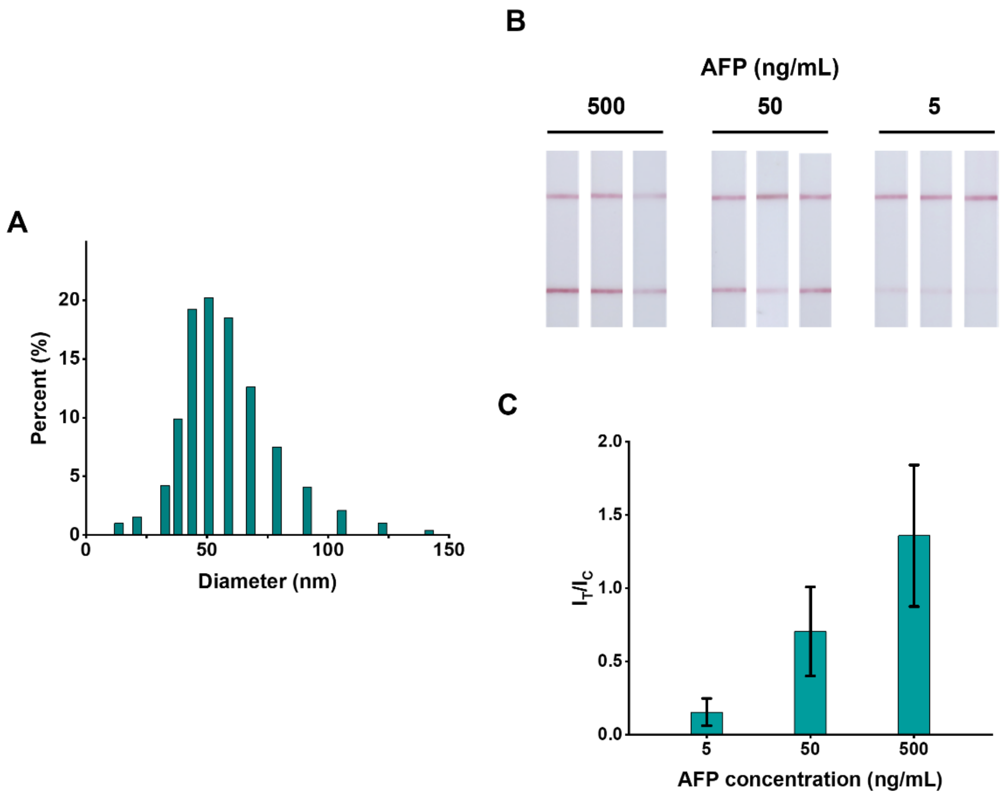

Fig. S26. Size effect studies of AA-AuNPs in LFIA. (A) The diameter distribution profile of large AA-AuNPs (~ 50 nm) measured by dynamic light scattering. (B) The testing strips of large AA-AuNPs LFIA responded to different concentrations of AFP (each group represents three duplicates). (C) The relevant intensity ratios of I_T/I_C. The error bars represent three duplicates.

Table S4. The recovery tests of AFP at different concentrations using AA-AuNPs LFIA.

Concentration (ng/mL)	Recovery (%)	Coefficient of variation (%)
2	84.22	3.49
10	89.41	3.05
100	110.7	3.84
1000	93.92	4.87

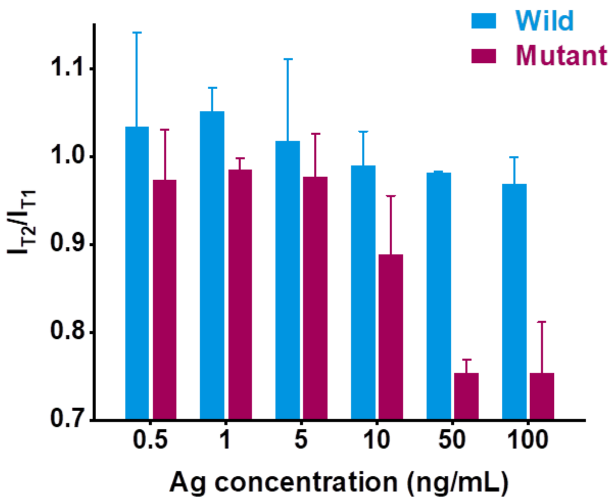

Fig. S27. AA-AuNPs LFIA for SARS-CoV-2. The intensity ratio signal (I_{T2}/I_{T1}) of AA-AuNPs LFIAs responding to different Ag concentrations of the nucleocapsid (N) protein of SARS-CoV-2 wild strain and B.1.1.7 mutant strain.

Movie S1.
Molecular dynamic simulation of IgG on the surface of Cit-loaded Au.

Movie S2.
Molecular dynamic simulation of IgG on the surface of AA-loaded Au.
